# Cytotoxic and Antifungal Amides Derived from Ferulic Acid: Molecular Docking and Mechanism of Action

**DOI:** 10.1155/2021/3598000

**Published:** 2021-11-01

**Authors:** Mayara Castro de Morais, Yunierkis Perez-Castillo, Valdenizia Rodrigues Silva, Luciano de Souza Santos, Milena Botelho Pereira Soares, Daniel Pereira Bezerra, Ricardo Dias de Castro, Damião Pergentino de Sousa

**Affiliations:** ^1^Laboratory of Pharmaceutical Chemistry, Department of Pharmaceutical Sciences, Federal University of Paraíba, 58051-900 João Pessoa, PB, Brazil; ^2^Escuela de Ciencias Físicas y Matemáticas, Universidad de Las Américas, Quito, Ecuador; ^3^Instituto Gonçalo Moniz, Fundação Oswaldo Cruz (IGM-FIOCRUZ/BA), Salvador, 40296-710 Bahia, Brazil; ^4^Laboratory of Experimental Pharmacology and Cell Culture, Department of Clinical and Social Dentistry, Federal University of Paraíba, 58051-900, Joao Pessoa, PB, Brazil

## Abstract

Amides derived from ferulic acid have a wide spectrum of pharmacological activities, including antitumor and antifungal activity. In the present study, a series of ten amides were obtained by coupling reactions using the reagents (benzotriazol-1-yloxy) tripyrrolidinophosphonium hexafluorophosphate (PyBOP) and *N,N*′*-*dicyclohexylcarbodiimide (DCC). All the compounds were identified on the basis of their IR, ^1^H- and ^13^C-NMR, HRMS data, and with yields ranging from 43.17% to 91.37%. The compounds were subjected to cytotoxic tests by the alamar blue technique and antifungal screening by the broth microdilution method to determine the minimum inhibitory concentration (MIC). The amides **10** and **11** displayed the best result in both biological evaluations, and compound **10** was the most potent and selective in HL-60 cancer cells, with no cytotoxicity on healthy cells. This amide had antifungal activity in all strains and had the lowest MIC against *Candida albicans* and *Candida tropicalis*. The possible mechanism of antifungal action occurs via the fungal cell wall. Molecular modeling suggested that compounds **10** and **11** interact with the enzymes GWT1 and GSC1, which are essential for the development of *C. albicans*. The findings of the present study demonstrated that compounds **10** and **11** may be used as a platform in drug development in the future.

## 1. Introduction

Ferulic acid (4-hydroxy-3-methoxycinnamic acid) is a natural organic compound abundantly distributed in fruits and vegetables [1]. It is well known potent phenolic antioxidant that has the property of scavenging free radicals and induce antioxidant enzymes, such as catalase and superoxide dismutase (SOD) [2, 3], in order to protect cellular macromolecules of peroxidation and oxidative damage. The presence of these characteristics has been depicted from numerous studies reported in the literature that show the pharmacological activity of ferulic acid in experimental models related to several pathologies, including diabetes, neurodegenerative diseases, and cardiovascular disease [4, 5]. Recent studies have demonstrated ferulic acid as a potential agent against some tumors [6]. This may be associated with its ability to eliminate reactive oxygen species (ROS) and stimulate cytoprotective enzymes [7], causing a decrease in lipid peroxidation, rupture of the double strand of DNA, inactivation of certain proteins, and disruption of biological membranes [8]. The stimulation of the detoxification enzyme seems to be another mechanism responsible for its anticarcinogenic action [9].

*Candida albicans* is an opportunistic microorganism that dramatically infects people with AIDS and with bone marrow transplantation. Other species, as *Candida tropicalis* and *Candida krusei*, are associated with the pathological process, especially for promoting the formation of biofilms and increasing drug resistance [10]. In fact, immunosuppression can predispose to *Candida* infection and induce the development of a spectrum of pathologies, compromising the quality of life and survival time of patients. Therefore, the development of new drugs is necessary to prevent infection by candidiasis/candidemia due to the emergence of *Candida* species resistant to current antifungals [11, 12].

Phenolic compounds such as ferulic acid are found in plants that have shown multiple health benefits, including protection against human infectious diseases [13, 14]. The bioavailability of this compound is decisive for its pharmacological properties. However, it has poor absorption, distribution, metabolism, and excretion (ADME) properties, with rapid phase II metabolic transformation (for example, methylation, glucuronidation, and sulfation), being excreted in the urine and feces. This leads to poor translation of resources in vitro for therapeutic applications in vivo [15, 16].

On the other hand, molecular modeling techniques are widely applied in the framework of drug discovery and development projects. Among structure-based techniques, molecular docking is rutinary applied for tasks such as virtual screening and the exploration of the binding mode of molecules to their receptors [17]. Another tool employed for structure-based modeling studies is molecular dynamic simulations. Molecular dynamics incorporates a more thorough description of the molecular interactions than that provided by molecular docking, with the consequent increase in computational complexity and the possibility to explicitly incorporate solvents and biological membranes into the modeling process. Tasks usually performed with molecular dynamics simulations include the investigation of the evolution of large biochemical systems in time, the study of the flexibility of biological systems, and the calculations of free energies of binding in a more accurate context than docking calculations [18].

These two methods are not exclusive to each other and can be combined, for example, molecular dynamics can provide a set of receptor conformations for their later use in molecular docking calculation incorporating receptor flexibility [19–21]. The growing computational processing capabilities have enabled the use of structure-based modeling tools at large scales and to incorporate molecular dynamics simulations in the postprocessing of molecular docking calculations. Specifically, the refinement of docking predictions with molecular dynamic-based methods has been recommended for discriminating correct from incorrect binding poses of a compound to its receptor [22, 23]. Furthermore, the postprocessing of docking predictions with free energy calculations derived from molecular dynamics increases the enrichment in virtual screening campaigns [24, 25]. Finally, combining molecular docking and molecular docking has been applied to the identification of the potential targets and binding models of bioactive compounds [26–29].

Despite the effectiveness of structure-based methodologies in drug discovery, these must be applied carefully, the obtained results must be carefully analyzed, and the limitations of the employed methods must be taken into account when interpreting the results [30]. Thus, the aim of this study was to prepare a series of amides derived from ferulic acid and evaluate their cytotoxic and antifungal activities. In addition, extensive molecular modeling studies, combining molecular docking and molecular dynamic simulations, were performed to investigate the potential mechanism of antifungal action.

## 2. Results and Discussion

### 2.1. Chemistry

**Scheme 1:**
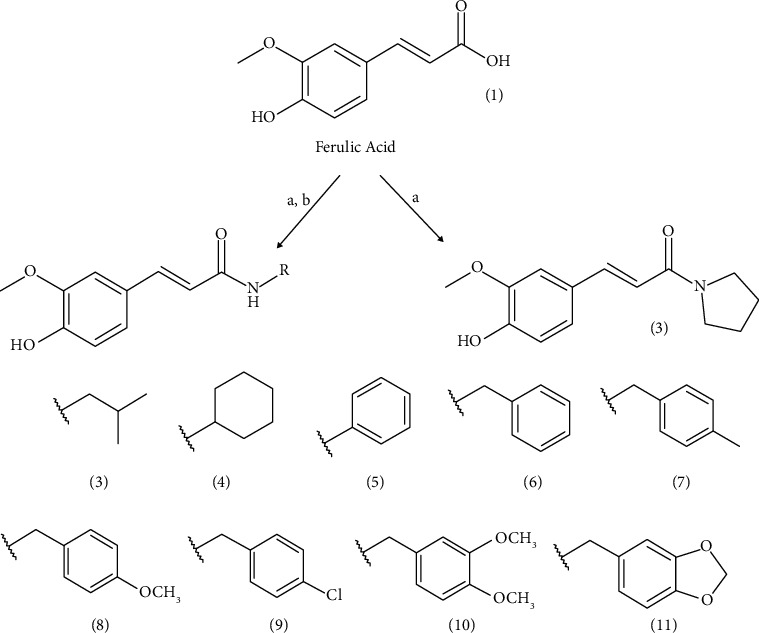
Reagents and conditions used to obtain the amides and structural formula. (a) DMF, Et_3_N, BOP, CH_2_Cl_2_, and 0°C to r.t. (b) DCC, DMAP, CH_2_Cl_2_, and r.t.

Ferulic acid (**1**) was used as the starting material for the preparation of a collection of ten amides that were obtained through coupling reactions with PyBOP (**2-7**) and DCC (**8-11**). The modifications were carried out by adding alkyl and aryl amines: isobutylamine (**2**), pyrrolidine (**3**), cyclohexylamine (**4**), phenylamine (**5**), benzylamine (**6**), 4-methylbenzylamine (**7**), 4-methoxylbenzylamine (**8**), 4-chlorobenzylamine (**9**), 3,4-dimethoxybenzylamine (**10**), and piperonylamine (**11**) (Scheme 1) [31, 32]. The compounds were characterized by infrared (IR) and nuclear magnetic resonance (NMR) spectroscopy, and in the case of the novel compounds **2** and 8, by high-resolution mass spectrometry (MALDI).

### 2.2. Cytotoxic Evaluation

The cytotoxic activity results of the ferulic acid derivatives against the growth of three human cancer cell lines and one healthy cell line are shown in Table 1. According to the results, considering compound **2** as the base skeleton of our study, a molecule unprecedented in the literature, it did not show cytotoxic activity for any cell line studied, suggesting that the presence of alkyl group does not contribute to this type of activity.

The compound **3** containing the pyrrolidine group did not show cytotoxic activity in the studied cell lines. Meanwhile, derivatives **4** with a cyclohexyl group and **5** with a phenyl group had selective activity in HL-60 cells with IC_50_ 68.42 *μ*mol/L and 50.40 *μ*mol/L, respectively. Although compound **3** has a substituent group with a volume similar to those of compounds **4** and **5**, the presence of nitrogen in the tertiary amide heterocyclic system resulted in cytotoxic inactivity. Compound **6**, which has a benzyl ring linked to methylene, showed cytotoxic activity against all cancer cells, with the best result against HL60 cells with IC_50_ 64.94 *μ*mol/L, but it showed cytotoxicity in MRC5 cells, demonstrating that the compound is not selective for tumor cells. Chavaria et al. 2019 [33] stated that compound **6** had a satisfactory antioxidant activity by test of ABTS (2,2′-azinobis (3-ethylbenzothiaziline-6-sulfonate)), IC_50_: 25.6 ± 1.8; and control: 18.2 ± 0.5. Compound **7** with the methyl group in the *para*-position of the benzyl ring was selective for HL-60 cells and had the IC_50_ reduced to 53.10 *μ*mol/L. Compound **8**, an unpublished molecule in the literature, showed cytotoxicity for the cell lines HCT-116, HL-60, and MRC-5, 64.65 *μ*mol/L, 70.27 *μ*mol/L, and 53.23 *μ*mol/L, respectively. However, it has low selectivity against tumor cells. It is worth mentioning here that compound **9** with a 4-chlorobenzyl group was selective for HL60 cells with IC_50_ 49.02 *μ*mol/L, suggesting that the presence of the electron withdrawing atom from the aromatic ring may have contributed to this effect. Compound **10** (dimethoxy substitution on the aromatic ring) had the best result with selectivity for the HL-60 tumor cell line and IC_50_ value of 36.45 *μ*mol/L. Meanwhile, the presence of the methylene dioxide radical in the **11** (IC_50_ 57.44 *μ*mol/L) did not contribute to the potentiation of cytotoxicity in the HL-60 tumor cells. Although synthetic derivatives have shown a lower cytotoxic potency than positive control (DOX), bioactive compounds can be used as structural models for chemical modifications in order to obtain new derivatives with a better cytotoxic profile. In addition, the synthesis of ferulic acid amides is of low cost when compared to most anticancer drugs, such as DOX.

### 2.3. Antifungal Evaluation

The antifungal activity (Table 2) of the ten amides was assessed via the minimum inhibitory concentration (MIC) which was determined using the microdilution methods against three species of *Candid*a strains (*Candida albicans, C. tropicalis*, and *C. krusei*). The bioactivity of the compounds was categorized as follows: (a) very strong bioactivity (MIC < 10 *μ*g/mL), strong bioactivity (MIC between 10 and 25 *μ*g/mL),good bioactivity (MIC between 26 and 125 *μ*g/mL), (d) moderate bioactivity (MIC from 126 to 500 *μ*g/mL), (e) mild bioactivity (MIC in the range of 501–1000 *μ*g/mL), and (e) absence of bioactivity (MIC > 1000 *μ*g/mL) [34].

Compounds **2**–**5** were found to be inactive. On the other hand, compounds **6**–**11** were found to be bioactive, with compounds **10** and **11** showing good bioactivity. The MFC/MIC ratio indicated that these compounds present fungicidal effects against all tested strains. As expected, the MIC and MFC values of nystatin were of 0.0043 *μ*mol/mL. The existence of CH_2_ between the group *R* and nitrogen may be contributing to the activity, considering that it is the difference observed between the groups of bioactive and inactive amides. Spacing gives the molecule more flexibility, increasing the number of conformers and thus increasing the likelihood of interaction between the analogue and its target [35]. Analogue **6** showed activity against the strains of *C. albicans* and *C. krusei* with a minimum inhibitory concentration (MIC) of 1.85 *μ*mol/mL and 0.82 *μ*mol/mL, respectively. Oliveira et al. [36] used vanillic acid as the starting material to prepare an amide containing the benzyl substituent, and it only showed activity against *C. albicans* with IC_50_ value 3.88 *μ*mol/mL. In contrast, analog **7** (with a 4-methylbenzyl group) showed activity against strains of *C. tropicalis* and *C. krusei*, with MIC of 1.68 *μ*mol/mL for both strains, but it was inactive against *C. albicans*. In another study, Oliveira et al. [36] showed that the 4-methylbenzylamide derived from vanillic acid has activity against the *C. glabrata* strain. This data shows the antifungal ability of **7**. While, in the present study, the amide **8** (with 4-chlorobenzyl substituent) was bioactive for all types of *Candida* strains (MIC = 1.59 *μ*mol/mL). In the amide **9**, the 4-methoxybenzyl group resulted in the inactivity of the compound against *C. albicans,* but it is bioactive for the other fungi. Table 2 shows that the presence of two methoxyls in the *meta-* and *para*-positions of the aromatic ring of the compound **10** (3,4-dimethoxybenzyl group), and a methylene dioxide group in the aromatic ring of compound **11** (piperonyl substituent) resulted in activity against all *Candida* strains studied. The lowest MIC values obtained were 0.18, 0.18, and 1.45 *μ*mol/mL and 0.19, 0.19, and 1.52 *μ*mol/mL, for *C. albicans, C. tropicalis*, and *C. krusei*, respectively. According to current research, oxygen in some structures provides a new center for hydrogen bonding that can influence the binding of the analog to the target site [35]. The compounds **10** and **11** were tested for prediction of their mechanism of action against the *C. albicans* strain using two pharmacological strategies, using ergosterol and sorbitol to determine likely activity on the plasma membrane or cell wall, respectively (Table 3).

Sterols participate in the constitution of all fungal cells. Ergosterol is the main sterol and modulates membrane fluidity, cell growth, and proliferation [37, 38]. Tests to detect the biological target of the title compounds **10** and **11** were performed by adding more ergosterol to the medium. No increase in the MIC of the compounds has been observed indicating that the fungus possibly does not act by inhibiting synthesis or by binding directly to ergosterol. Azoles and polyenes are well known classes of antifungal drugs that act on ergosterol to treat fungal infections [39].

Protoplasts stabilized with osmoprotectors have been important biochemical tools to study the architecture of the cell wall [40]. Moreover, osmotic stability has been used with *C. albicans* and other fungi to study the mechanism of action of some antibiotics [41, 42]. Damage to essential cell wall components from antifungal agents (inhibitors of cell wall synthesis) would lyse cells in the absence of an osmoprotectant; however, cells will continue to grow if a suitable stabilizer is present in the medium. The test with sorbitol, an osmotic protector, and the MIC of title compounds **10** and **11** increased, with growth, indicating that the substance acts interfering cellular functions that involve the participation of the cell wall. The results of the study reveal that in the presence of sorbitol, the fungus is protected and continues to reproduce. The substance thus acts by modulating the function of the cell wall [43, 44].

### 2.4. Molecular Modeling

Compounds **10** and **11** were docked into the binding sites of the 19 targets listed in Table 4 as described in the Material and Methods section. The top scored conformer per target is presented in Table 5, and the full results of the molecular docking calculations are provided as Supplementary Materials in Table S1.

**Figure 1:**
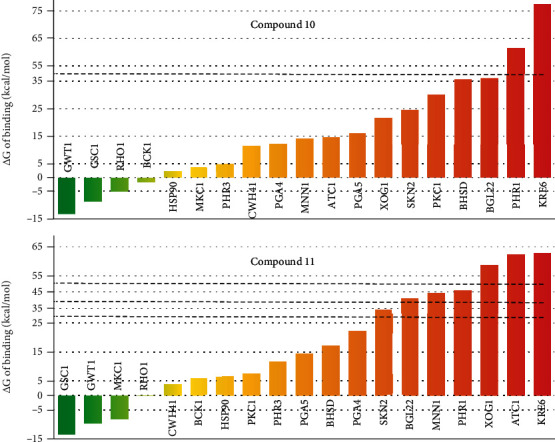
Predicted free energies of binding of compounds 10 (top) and 11 (bottom) to its potential targets. Targets are ranked from most favorable energies (green) to less favorable values (red). Dashed lines are used to indicate cuts on the figure for a better visualization.

The docking calculations lead to 37 possible solutions predicted for each compound, totaling 74 complexes to be further examined. For most of the studied proteins, more than one possible binding pose is identified, and the visual inspection of the predicted binding poses reveals meaningful interactions between the compounds under investigation and their potential targets. The scaling and aggregation of the scores presented in Table 5 reveal that BHSD, SKN2, RHO1, and GWT1 are the top scored targets for both compounds. It must be considered that during molecular docking several factors involved in molecular recognition are neglected. This is a known limitation of all docking software which is also necessary for the prediction of potential ligand-receptor complexes of large amounts of compounds in a reasonable time. As previously shown, molecular docking can be effective in the initial identification of possible binding modes of ligands to receptors. However, the estimation of the free energies of binding from ensembles of molecular complexes conformations using more accurate modeling approaches such as MD can aid in the identification of feasible complex [22, 45–47]. To reduce the number of possible targets of compounds **10** and **11**, their predicted complexes with the potential receptors listed in Table 5 were subject of MD simulations and MM-PBSA calculations as described in the Material and Methods section. The total MD simulation time accounted for 1.48 *μ*s. The estimated free energies of binding of all predicted complexes are provided as Supplementary Materials in Table S2 and summarized in Figure 1. Only the ligand pose providing the lowest (best) free energy of binding per target is presented in Figure 1 and discussed from here on.

Among the explored potential targets, the membrane proteins GWT1 and GSC1 stand above all in terms of free energies of binding for both compounds. The results derived from the MD simulations contrast with those obtained with the molecular docking calculations. In fact, the top two targets providing the best docking scores (BHSD and SKN2) position among the 50% worst ranked proteins for the two compounds when the more accurate MD simulations studies are performed. Overall, the complexes GSC1-**11** and GWT1-**10** have very similar free energies of binding, with values of -13.38 kcal/mol and -13.18 kcal/mol, respectively. Interestingly, GWT1 ranks as the most probable target of compound **10**, while GSC1 ranks in the first position for compound **11**. Based on the results of the free energies of binding calculations, we propose that the main mechanism of action of this series of compounds interfering with the cell wall integrity in *C. albicans* is likely through their binding to the GWT1 and GSC1 receptors.

The RMSD of compounds **10** and **11** along the MD simulations of the predicted complexes with GWT1 and GSC1 relative to the docking solutions was analyzed. The plots of RMSD vs. MD snapshot for all complexes are provided as Supplementary Materials in Figures S33 and S34. In all cases, the RMSD values are lower than 2 Å, indicating that ligands are stable during our simulations. It is worth noting that there are differences in these plots for the same complex. This is a consequence of setting different random initial velocities during the five MD simulations performed for each docking complex. By exploring different conformational states, close to the docking predicted binding mode, it is possible to obtain a more diverse set of complex conformations for the prediction of the free energies of binding compared to the use of a single MD trajectory.

**Figure 2:**
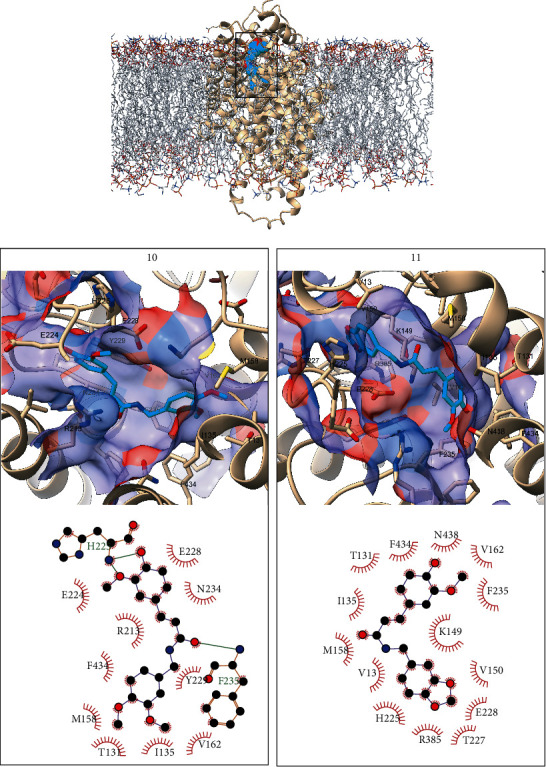
Overall orientation of compounds **10** and **11** bound to GWT1 (top) with the ligands represented as cyan spheres, the receptor as tan ribbons, and the membrane as light gray sticks. The detailed binding modes of compounds **10** and **11** are provided in the left and right bottom boxes, respectively. The receptor is colored tan, and the ligand cyan and heteroatoms are colored as red (oxygen), blue (nitrogen), and yellow (sulfur). In the interaction diagrams, all atoms are represented only for residues forming hydrogen bonds (dashed lines) with the ligand.

**Figure 3:**
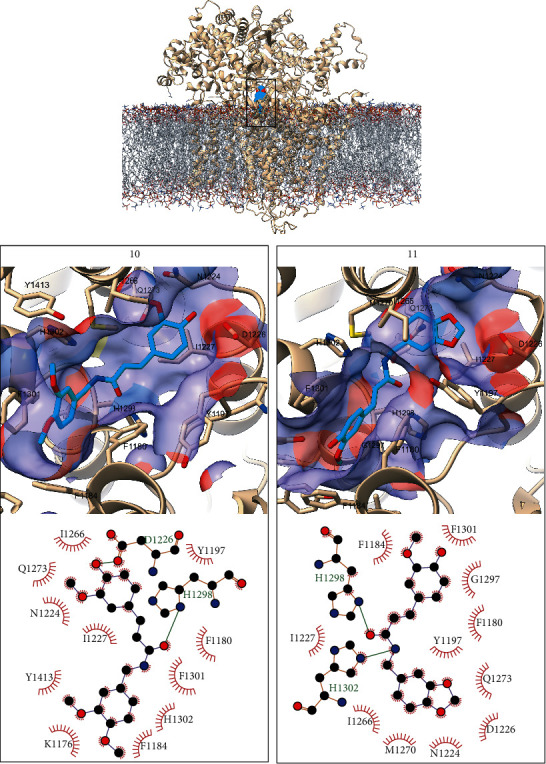
Overall orientation of compounds **10** and **11** bound to GSC1 (top) with the ligands represented as cyan spheres, the receptor as tan ribbons, and the membrane as light gray sticks. The detailed binding modes of compounds **10** and **11** are provided in the left and right bottom boxes, respectively. The receptor is colored tan, and the ligand cyan and heteroatoms are colored as red (oxygen), blue (nitrogen), and yellow (sulfur). In the interaction diagrams, all atoms are represented only for residues forming hydrogen bonds (dashed lines) with the ligand.

Figures 2 and 3 represent the predicted binding modes of compounds **10** and **11** to the GWT1 and GSC1 receptors as well as the predicted ligand-receptor interactions. The depicted conformations of the complexes correspond to the centroid of the most populated cluster resulting from clustering the ligand poses present in the 100 MD snapshots used for MM-PBSA calculations. Only the interactions observed in at least 50% of the analyzed MD snapshots are represented in the interaction diagrams, and the same rule is followed for labeling the residues in the representation of the ligand binding modes. The analyses of the frequencies of interaction were performed with UCSF Chimera 1.15 [48] and Cytoscape 3.8.2 [49], the interaction diagrams were produced with LigPlot+2.2 [50], and figures representing molecular structures were generated with ChimeraX 1.1 [51].

In the predicted binding modes of compounds **10** and **11** to GWT1 the ligands present different orientations within the cavity. Taking the common hydroxy-methoxyphenyl moieties as references, that of **10** points to the entrance of the cavity while for **11,** it locates at the bottom of the binding pocket. A shared feature between both complexes is that the dimethoxyphenyl group of **10** and the hydroxy-methoxyphenyl substituent of **11** overlap at a hydrophobic region lined by T131, I135, M158, V162, F235, and F434 at the bottom of the cavity. This suggests that this region could be important for the stability of the predicted complexes. Other interactions observed in the two complexes, despite their different natures, are with H225 and E228. In the case of **10**, it hydrogen bonds the backbone of H225 while the benzodioxol group of **11** stacks parallel to the same amino and makes extensive contacts with V13, K149, T227, and R385. The difference in the free energies of binding between both complexes, -13.18 kcal/mol and -9.55 kcal/mol for **10** and **11**, respectively, can be explained by the two hydrogen bonds observed for **10** with H225 and F235 in contrast to the lack of this type of interactions for compound **11**. This different hydrogen bonding pattern emerges as a consequence of the different binding modes predicted for the two compounds. The possible binding of compound **11** in a conformation similar to that obtained for **10,** that is energetically more favorable, is discussed below in this section.

As in the complexes predicted for GWT1, the two compounds are predicted to orient differently in the GSC1 binding pocket. For this receptor, the predicted binding poses are rotated 180° relative to each other, leading to 85% of the interacting receptor amino acids being the same for both ligands. As shown in Figure 3, the carbonyl group of the ligands is predicted to hydrogen bond H1298. Additional hydrogen bonds are predicted between compound **10** and D1226 as well as between compound **11** and H1302. Less frequent (in less than 50% of the analyzed MD snapshots) hydrogen bond interactions are predicted between **10** and H1302 and between **11** and N1224. One interaction proposed to contribute to the stability of the predicted complexes is the stacking of the dimethoxyphenyl group of **10** and the hydroxy-methoxyphenyl substituent of **11** parallel to F1180 and F1301 and perpendicular to F1184. On the other extreme of the ligands, the hydroxy-methoxyphenyl and benzodioxol moieties of **10** and **11** are predicted to bind in a region defined by Y1197, N1224, D1226, I1227, I1266, and Q1273.

To further assess the modeling results, one known inhibitor of each of the GWT1 and GSC1 proteins in *C. albicans* was extracted from the ChEMBL database and subjects to the same modeling strategy applied to compounds **10** and **11**. The selected benchmarking compounds were the GWT1 inhibitor CHEMBL4475362 and the GSC1 inhibitor CHEMBL1770508. A visual analysis of the predicted poses of the reference compounds reveals that they bind GWT1 and GSC1 in the same regions as compounds **10** and **11**, overlapping their geometries. The results of these calculation show that the predicted free energy of binding for the CHEMBL4475362-GWT1 complex is -15.47 kcal/mol, while for the CHEMBL1770508-GSC1 complex is Δ*G* = −4.12 kcal/mol. According to these results, the predicted **10**-GWT1 complex has a free energy of binding close to that predicted for the benchmarking system, while the difference between the lather and the **11**-GWT1 complex is larger. This indicates that compound **10** could be a good initial candidate for the development of *C. albicans* GWT1 inhibitors. On the other hand, the 10-GSC1 and 11-GSC1 complexes provided lower (better) free energies of binding that the benchmark system, suggesting an improved inhibitory activity.

When analyzing the presented results, it is intriguing that the predicted binding modes of the two highly similar compounds to GWT1 and GSC1 do not superimpose. It must be taken into account that the complexes for MD simulations were obtained from molecular docking calculations. None of the most probable binding modes of compound **11** to GWT1 overlap with the predicted pose of ligand **10** having lower free energy of binding, and the same occurs for the GSC1 receptor. Although striking, this is not surprising given the limitations of molecular docking algorithms to explore the flexibility of the receptor and the simplicity of their treatment of molecular interactions compared to MD simulations. To explore if poses of compounds **11** bound to GWT1 and **10** bound to GSC1 similar to those observed in the **10**-GWT1 and **11**-GSC1 complexes are possible, molecular docking was repeated imposing the ligand conformations observed in the later complexes as constraints. Since docking calculations are configured to explore the flexibility of the receptors' side chains, the selected constraints would produce binding modes resembling those present in the **10**-GWT1 and **11**-GSC1 complexes.

**Figure 4:**
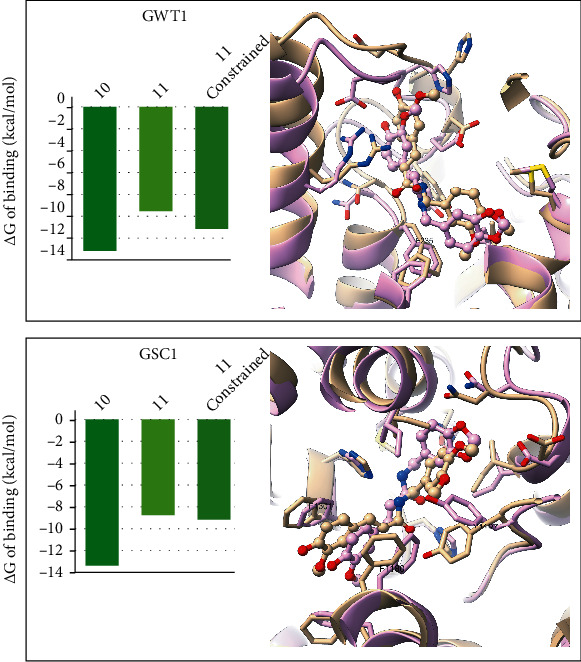
Modeling of compound **11** bound to GWT1 constrained to overlap with the **10**-GWT1 complex (top) and of compound **10** bound to GSC1 constrained to overlap with the **11**-GSC1 complex (bottom). Complexes containing compound **10** and **11** are colored in tan and purple, respectively. The ligands are shown using a ball and stick representation.

The constrained docking calculations produced possible binding modes of compound **11** to GWT1 and of **10** to GSC1 overlapping with the reference complexes. These were subject to the same MD simulations and MM-PBSA calculations' protocol applied to the rest of the complexes studied herein. The results of these calculations are summarized in Figure 4, showing that the docking constrained **11**-GWT1 complex produces better free energy of binding (-11.14 kcal/mol) than the previously analyzed model (-9.55 kcal/mol). Still, this new **11**-GWT1 model possesses a higher value of free energy of binding than the reference **10**-GWT1 complex that can be explained from the subtle structural differences between compounds **10** and **11**.

Our previous analyses suggest that a hydrophobic region at the bottom of the binding cavity delimited by T131, I135, M158, V162, F235, and F434 could be important for the stabilization of the complexes with GWT1. In the presented model, one of the methoxy substituents in the dimethoxyphenyl group of **10** projects toward V162, F235, and F434. In the docking constrained model, the benzodioxol group of compound **11** overlaps with the dimethoxyphenyl moiety of **10**. Being the dimethoxyphenyl substituent larger than the benzodioxol one, compound 11 must go deeper into the binding site to maintain its interaction with V162, F235, and F434. This displacement has two main consequences; firstly, the flexible loop containing H225 moves closer to the binding pocket, and this residue maintains the hydrogen bond with the ligand. Secondly, the position of the carbonyl group in **11** changes relative to the predicted binding mode of compound **10**, reducing the probabilities of hydrogen bonding between this group in compound **11** and the backbone of F235. The loss of this interaction might explain the reduced free energy of binding in the docking constrained **11**-GWT1 model compared to the **10**-GWT1 complex.

For GSC1, the predicted free energy of binding in the model containing the constrained conformation of compound **10** (-9.17 kcal/mol) shows a marginal improvement relative to the previously analyzed **10**-GSC1 complex (-8.76 kcal/mol). In contrast to the subtle structural changes observed in the constrained **11**-GWT1 model, the structural differences between the dimethoxyphenyl and benzodioxol rings have a high impact on the binding to GSC1, to accommodate one of the methoxy substituents in the dimethoxyphenyl group of **10** Y1197 rotates 90° relative to its position on the **11**-GSC1 model. This rotation takes place in direction to the position originally occupied by F1180, displacing it. The movement of F1180 reaccommodates the positions of the ligand's hydroxy-methoxyphenyl group and F1301 to maintain the stacking of the ligand aromatic moiety between F1180 and F1301. The slight displacement of the ligand in the docking constrained **10**-GSC1 model relative to the **11**-GSC1 one also makes the frequency of hydrogen bonding with H1298 and H1302 lower compared to the later model.

According to the above discussed results, the binding of compounds **10** and **11** to GWT1 with a similar binding mode is plausible, despite the docking methodology does not provides high scored conformers of **11** overlapping with **10**. For GSC1, the difference in free energies of binding between the docking predicted pose of **10** and the binding mode produced by the constrained mode is small. Thus, any of the two **10**-GSC1 models studied (nonconstrained and constrained) are possible within the framework of the applied modeling approach. Even though the possible binding of similar compounds to their target in nonoverlapping conformations could be interpreted as a deviation on the SAR for a series of molecules, different examples of small structural modifications leading to completely different binding modes have been reported in the literature [52–55].

Our models suggest that the presence of the CH_2_ group between the amide nitrogen and the *R* group is essential for compounds **6**-**11** to be fully complementary with the GWT1 and GSC1 receptors. The same requirement can also apply to the size of the *R* group, only the bulkiest substituents such as those present in compounds **10** and **11** are able to completely exploit the complementarity with the receptors' shapes. Moreover, maintaining the right size of the ligands contributes to positioning the central linker of the compounds favorably to hydrogen bond the receptors as shown in Figures 2 and 3.

The analyses presented hitherto, including those of the docking constrained models, suggest that compounds **10** and **11** have the potential to be dual GWT1-GSC1 inhibitors. Specifically, compound **10** could better inhibit GWT1 than GSC1 while **11** could be a better inhibitor of GSC1. These two enzymes have been shown to be essential for the development of *C. albicans* [56, 57]. GWT1 catalyzes the acylation of the inositol, and its inhibition affects the integrity of the cell wall, producing cell growth defects [58, 59] and reducing the adherence of the fungus to the host cell. The role of GSC1 in *C. albicans* has been linked to the synthesis of *β*-1,3-glucan which is the main polysaccharide in the cell wall, thus, disrupting GSC1 functioning leads to cell death [60]. In consequence, the predicted inhibition of GWT1 and GSC1 by compounds **10** and **11** could explain their observed antifungal activity.

When interpreting the results provided by the modeling methodology applied in this study, it must be considered that these lead to hypotheses that can guide and optimize future experimental research. In other words, modeling studies cannot provide a definitive answer to the mechanism of action of the studied compounds, but narrows and prioritizes the hypothesis with the best chances to provide a successful experimental outcome. Hence, we consider the modeling results a valuable tool to design future experimental investigations on determining the mechanism of action and optimizing of the antifungal activity of the series of ferulic acid derivatives herein investigated.

## 3. Materials and Methods

### 3.1. General Information

The ^1^H and ^13^C-NMR and IR signals assigned to derivatives of **1** were comparing with signals already published. For unpublished compounds (**2** and **7**), high resolution mass spectrometry was performed using LS-MALDI TOF/TOF to confirm the synthesis.

The derivatives were purified by column adsorption chromatography (CC) using silica gel 60 (ART 7734—Merck-Millipore, St. Louis, MO, USA). Infrared spectra were performed using FTIR spectrophotometry; The ^1^H and ^13^C-NMR spectra were obtained using a Bruker Ascent™ instrument (Bruker, Bremen, Germany) operating at 200 and 50 MHz.

### 3.2. General Synthesis of Amides **2**–**6**

In a round-bottom flask, ferulic acid (0.1 g, 0.51 mmol) was dissolved in DMF (1.02 mL) and trimethylamine (0.068 mL, 0.51 mmol). The solution was cooled in an ice water bath, and the appropriate amine (0.51 mmol) was added, followed by a solution of BOP (0.51 mmol) in CH_2_Cl_2_ (1.02 mL). The mixture was stirred at 0°C for 30 min and then at room temperature for 3 h. After of the removal of CH_2_Cl_2_ under reduced pressure, the solution was diluted with ethyl acetate (10 mL) and transferred to separatory funnel containing water (10 mL). The products were extracted with ethyl acetate (10 mL) three times. The organic phase was washed successively with 1 N HCl, water, 1 M NaHCO_3_ and water, dried over Na_2_SO_4_, and evaporated. The residue was purified on a silica gel 60 column chromatography (eluent: hexane-ethyl acetate, 7 : 3) [61]. Spectroscopic data for the compounds in this study are available in the Supplementary Materials (available here).

### 3.3. General Synthesis of Amides **7**–**11**

Ferulic acid (0.51 mmol), 4-dimethylaminopyridine (0.051 mmol), and amine (0.51 mmol) were added to a solution of dicyclohexylcarbodiimide (0.51 mmol) dissolved in CH_2_Cl_2_ (3 mL). The solution was stirred for 24 to 48 h at room temperature. The solvent was evaporated under reduced pressure. After adding water to the residue, extraction was done with ethyl acetate (3 × 10 mL). The organic phase was treated with 1 N hydrochloric acid solution (10 mL) and sodium bicarbonate 5% solution (10 mL), followed by water (10 mL). The organic phase was dried with anhydrous Na_2_SO_4_ and the solvent evaporated under reduced pressure. The residue was purified by silica gel column chromatography (eluent: hexane-ethyl acetate, 7 : 3) to obtain the described compounds [36]. Spectroscopic data for the compounds in this study are available in the Supplementary Materials (available here).

### 3.4. Cytotoxicity Test

#### 3.4.1. Cells

HepG2 cells (human hepatocellular carcinoma), HCT116 (human colon carcinoma), HL-60 (human promyelocytic leukemia), and MRC5 (human lung fibroblast) obtained from the ATCC (Manassas, VA, USA) were used. Cells were cultured in cell culture bottles (75 cm 3, 250 mL volume), and media used were RPMI 1640 and supplemented with 10% fetal bovine serum. Cells were maintained in incubators with 5% CO_2_ atmosphere at 37°C. Cellular growth was monitored daily with the use of an inversion microscope. The medium was changed whenever cell growth reached the necessary confluence for nutrient renewal. For the maintenance of adhered cells, trypsin (0.25%) was used for the cells to detach from the walls of the bottles. Cell cultures showed microplasma negatives, as judged by placement with Hoechst (Mycoplasma Stain Kit, Cat. MYC1, Sigma-Aldrich, St. Louis, MO, USA).

#### 3.4.2. Cytotoxicity Assay

To assess the cytotoxicity of the substances, the alamar blue test was performed after 72 h of exposure with the test substances. Alamar blue, known as resazurin [62], is a fluorescent/colorimetric indicator with redox properties. The reduction in alamar blue reflects cell proliferation. This was initially used to indicate cell growth and/or viability in monitoring lymphocyte proliferation [63] and currently has several applications. Initially, cells were plated in 96-well plates (100 *μ*L/well of a solution of 0.3 × 106 cells/mL for cells in suspension and 0.7 × 105 cells/mL for adhered cells). After 24 h of incubation, the test substances dissolved in DMSO were added to each well and incubated for 72 h. Doxorubicin was used as a positive control. The negative control received the same amount of DMSO. Four hours before the end of the incubation period, 20 *μ*L of the stock solution (0.312 mg/mL) of alamar blue (resazurin) was added to each well. Absorbances were measured at wavelengths of 570 nm (reduced) and 600 nm (oxidized) using a plate reader [35].

### 3.5. Antifungal Test

#### 3.5.1. Microorganisms

Antifungal activity evaluations were performed using references strains of *Candida* spp., obtained from the Centraalbureau voor Schimmelcultures (CBS, Ultrecht, Netherlands): *Candida albicans* CBS 562, *Candida tropicalis* CBS 94, and *Candida krusei* CBS 573. Nystatin, Tween 80%, DMSO, caspofungin diacetate, and ergosterol were obtained from Sigma-Aldrich® (St. Louis, MO, EUA) and sorbitol (D-sorbitol anhydrous) from INLAB® (São Paulo, Brazil). All assays were performed in triplicate in three independent experiments. The culture medium used for maintenance of the microorganisms was Agar Sabouraud Dextrose (ASD). Inoculants were adjusted to a final concentration of 2.5 × 10^3^ CFU/mL (Clinical and Laboratory Standards Institute, 2002). All assays were performed in triplicate in three independent experiments.

#### 3.5.2. Determination of the Minimum Inhibitory Concentration (MIC) of the Tested Compounds

MIC was determined by the microdilution technique, as previously described by the adapted Clinical and Laboratory Standards Institute (CLSI, 2002) method [64]. The compounds were subjected to the microdilution technique in 96-well plates, with U bottom. The samples were dissolved with DMSO and sterile distilled water (up to 1.0 mL). Through serial dilutions, concentrations of the evaluated compounds ranging from 1000 *μ*g/mL to 7.8 *μ*g/mL. The culture sterility medium, the evaluated substances, and the microbial growth were carried out in parallel. The plates were closed and subjected to a temperature of 35 ± 2°C for 24 h. 1% TTC (2,3,5-triphenyl tetrazolium chloride, Sigma-Aldrich®, St. Louis, MO, EUA) was added to each well to confirm the presence of viable microorganisms [65]. MIC was defined as the lowest concentration of the test substance that inhibits visible microbial growth.

#### 3.5.3. Determination of the Minimum Fungicide Concentration (MFC) of the Tested Compounds

The minimum fungicide concentration (MFC) of the compounds was obtained after the interpretation of the MIC. Three (3) aliquots of 30 *μ*L of supernatant were removed from the wells (where complete inhibition of fungal growth was analyzed) and placed in Petri dishes containing 15 mL of agar Sabouraud dextrose. The plates were incubated at 35 ± 2°C for 24 h for visual counting of colony forming units [66, 67]. To determine whether the compounds had fungistatic (MFC/MIC ≥ 4) or fungicidal (MFC/MIC < 4) activity, the MFC/MIC ratio was calculated [67].

#### 3.5.4. Mechanism of Antifungal Action for Amides

*(1) Sorbitol Assay*. The microdilution technique was performed in the presence of sorbitol (D-sorbitol, anhydrous) (INLAB laboratory), to determine the mode of action of the compounds on the cell wall of *C. albicans* CBS 562. For this test, the inoculum was prepared with sorbitol to a final concentration of 0.8 M. The plates were incubated at 35 ± 2°C, and readings were taken 24 h and 48 h after incubation. Caspofungin was used as positive control at an initial concentration of 5 mg/mL [68–70].

*(2) Ergosterol Test*. The test of the compounds was performed using the microdilution technique, as previously described, in the presence of exogenous ergosterol at 400 *μ*g/mL. Nystatin was used as positive control. The plates were incubated at 35 ± 2°C, and readings were taken at 24 and 48 h [40–42].

### 3.6. Molecular Docking

According to the experimental results, proteins related to the synthesis and maintenance of the cell wall in *C. albicans* were selected for modeling studies. The targets were selected according to the information available in the scientific literature as well as from the Candida Genome Database and are listed in Table 4 [71–77]. From these, only two proteins had three dimensional structures deposited at the Protein Data Bank database, XOG1, and HSP90 [78]. For the rest of the selected proteins, homology models were generated with the Swiss model server [79]. Different homology models were obtained for the target sequences and, among them, the one with the higher QMEAN4 score was selected as the best one for each target. Considering that values of the QMEAN4 score lower than −4 are indicative of low-quality models, and the structural models of sequences for which no model fulfilling this criterion could be obtained were retrieved from the AlphaFold Protein Structure Database deposited at the EMBL-EBI (https://alphafold.ebi.ac.uk/) [80]. The UniProt accessions, IDs used along the manuscript, targets' description, and source of the protein structural models are presented in Table 4. The table also includes the PDB template, target coverage by the template and QMEAN score of the employed homology models. The initial 3D structures of the compounds were obtained with the OpenEye's Omega version 3.1.1.2 software [81], and AM1-BCC charges were added to its most stable conformer with OpenEye's Molcharge version 2.0.1.2 [82].

The Gold software version 2021.1.0 [83] was used for molecular docking of compounds **10** and **11** to their potential targets following the protocol described in our previous publication [45]. In brief, all water and noncatalytic cofactors were removed from the receptors. Cocrystallized ligands in the receptor or in the templates used for homology modeling were used to define the ligands' binding pocket. Any residue within 6 Å of the reference ligand was considered for the binding cavity, and the detect cavity option of gold was turned on. For proteins whose models were obtained from the AlphaFold repository, reference ligands were obtained from low homology proteins having a similar function and sharing the same folding. In two cases, GWT1 and GSC1, no reference ligand could be defined, and the binding pockets were manually defined from mutagenesis and functional data available in the literature [84, 85]. The list of X-ray structures from which reference ligands were extracted to define the binding pockets of all proteins but GWT1 and GSC1, as well as the residues selected for the binding cavity of the later proteins, is provided as Supplementary Materials in Table S3.

The ChemPLP scoring function was selected for primary docking with ligand flexibility set to very flexible (200% Search efficiency). The number of explored binding modes was set to 30, and the resulting binding poses were rescored with the GoldScore, ChemScore, and ASP scoring functions of gold.

The selection of the most probable binding modes of **10** and **11** to the explored targets was carried out following the same approach as in [30]. Given a score *S*_*i*,*j*_ for conformer *C*_*i*_ according to the scoring function *S*_*j*_, its scaled score *Z*_*i*_ was computed as

(1) $$\begin{array}{c} Z_{i}=\frac{\sum_{j}\left(S_{i,j}-\bar{S_{j}}/std\left(S_{j}\right)\right)}{4}, \end{array}$$

where $\bar{S_{j}}$ is the mean of the scoring function *S*_*j*_ across all conformers, and std(*S*_*j*_) is the standard deviation of the *S*_*j*_ values. Conformers with *Z*_*i*_ > 1 were selected as possible binding modes of compounds **10** and **11** to the targets under investigation and selected for further investigations.

### 3.7. Molecular Dynamics and MM-PBSA Calculations

Amber 20 [86] was employed for molecular dynamic (MD) simulations. The ff19SB and gaff2 force fields were chosen for proteins and nonamino acidic residues, respectively. The same modeling protocol was applied to the predicted complexes, except to those involving the membrane proteins GSC1 and GWT1. This protocol included two energy minimization steps, heating, equilibration, and the production runs. MD simulations took place in explicit solvent and otherwise noted default parameters were employed.

For all complexes but those predicted with GSC1 and GWT1, the topology and corresponding forcefield modifications of compounds **10** and **11** were obtained with antechamber. A truncated octahedron box was constructed around the systems that were solvated with OPC water molecules. The excess charges on the systems were neutralized through the addition of either Na^+^ or Cl^−^ ions. In all stages of simulation, the PME method was used to treat long range electrostatic interactions. The PME cutoff distance was set to 12 Å for the first energy minimization step and to 10 Å for the rest of the MD stages listed above.

The solvated and neutralized systems were minimized for 500 steps of the steepest descent method followed by 500 cycles of conjugate gradient at constant volume. During this first minimization stage, a force constant of 500 kcal/mol·Å^2^ was used to restrain all atoms except water molecules and ions. The minimized system was then subject of the second minimization stage consisting in 1500 steps of the steepest descent method followed by 1000 cycles of conjugate gradient at constant volume with no restrains.

Afterward, the system was heated from 0 to 300 K at constant volume. All atoms except water molecules and ions were restrained with a force constant of 10 kcal/mol·Å^2^ during heating. Heating was performed for 10,000 steps with a time step of 2 fs. From this stage on, a Langevin thermostat with a collision frequency of 1.0 ps^−1^ was employed, and the SHAKE algorithm was used to constrain the bonds involving hydrogen atoms. The heated system was then equilibrated at constant pressure of 1 bar and 300 K. Pressure was controlled using isotropic position scaling with a relaxation time of 2 ps during equilibration. The last snapshot derived from the equilibration process was used as input for 5 different MD simulations of 4 ns length each. Different initial velocities were assigned to each atom in all these MD simulations.

The complexes involving GSC1 and GWT1 were prepared with the CHARMM-GUI web server [87, 88]. Systems containing the larger GSC1 protein were embedded in a lipid bilayer formed by 100 1-palmotoyl-2-oleoylglycero-3-phosphocholine (POPC), 100 1-palmitoyl-2-oleoylphosphatidylethanolamine (POPE), and 50 cholesterol (CHL) molecules on each side. On the other hand, the membrane for the complexes having the smaller GWT1 receptor was formed by 50 POPC, 50 POPE, and 25 CHL lipids per side. The systems containing the receptor, the ligand, and the lipids bilayer were solvated and neutralized with OPC water molecules and 0.15 M of KCl, respectively. As for the soluble proteins, the Amber ff19sb and gaff2 force fields were selected to parametrize the proteins and ligands, respectively. The energy minimization, heating, and equilibration steps for these systems were conducted using the configuration files provided by the CHARMM-GUI server. Production runs proceeded as for the rest of the studied complexes.

The free energies of binding of the complexes were estimated by means of MM-PBSA calculations performed with Amber Tools 20 [86]. From each of the five production runs, 20 snapshots were evenly selected from the 1 ns to 4 ns simulation time interval. In this way, 100 snapshots were obtained for free energy of binding calculations with the MMPBSA.py program. For MM-PBSA calculations, the ionic strength was set to 150 mM, and default implicit solvent parameters were set. For the complexes containing membrane proteins, a heterogeneous dielectric implicit membrane model (memopt = 3) was selected for MM-PBSA calculations, and the solute dielectric constant was set to 2. In addition, the thickness of the membrane was set to the average distance between the N31 atoms of the lipids. The center of the membrane was determined as the average value of the *Z*-coordinates of the later set of atoms. Both the thickness and center of the implicit membrane were computed from the total 20 ns simulation time of each complex.

## 4. Conclusions

Synthesis and biological activities of a set of some amide ferulic acid derivatives **2**–**11** were reported. The study showed that modifications in the 3-hydroxy-4-methoxycinnamic structure may reveal new bioactive compounds, mainly against the cancer cell HL-60, where most compounds (**4**–**11**) showed cytotoxic activity. Compound **10** had the best inhibitory action against HL-60 cell proliferation and did not show any cytotoxicity against healthy cells (MRC5). Regarding the antifungal potential, it was possible to observe that all derivatives with a spacer (CH_2_) between the group *R* and NH (derivatives **6**–**11**) were bioactive. However, compound **10** was found to be more effective probably by causing damage to essential components of the cell wall resulting in lysis of the fungal cell. Results of the molecular simulations study performed with the two best antifungal amides revealed significant interactions between the compounds under investigation and their possible biological targets. It has been suggested that compound **10** and **11** interact with the enzymes GWT1 and GSC1, which are essential for the development of the fungus. The main residues involved in the interaction of the compounds with GWT1 were determined as T131, I135, M158, V162, H225, E228, HF235, and F434. For GSC1, the binding mechanism was dominated by interactions with F1180, F1184, H1298, F1301, and H1302. It must be highlighted that the structure-based modeling of the GWT1 and GSC1 proteins is possible since very recently after protein models have been produced with the AlphaFold algorithm. In addition, amides derived from ferulic acid are obtained in a single reaction step and at a lower production cost, when compared to most antifungal drugs, such as nystatin.

## Figures and Tables

**Table 1:** Cytotoxic activities of ferulic acid-derived amides against the growth of human cancer lines and healthy cell line.

Amides	HCT116		HEPG2		HL60		MRC5	
	*μ*g/mL	*μ*mol/L	*μ*g/mL	*μ*mol/L	*μ*g/mL	*μ*mol/L	*μ*g/mL	*μ*mol/L
**2**	>25	>100.27	>25	>100.27	>25	>100.27	>25	>100.27
**3**	>25	>95.73	>25	>95.73	>25	>95.73	>25	>95.73
**4**	>25	>90.79	>25	>90.79	18.84 (11.58–27.65)	68.42 (42.05–100.42)	>25	>90.79
**5**	>25	>88.23	>25	>88.23	14.28 (9.40–21.70)	50.40 (33.17–76.59)	>25	>88.23
**6**	18.93 (13.48–26.57)	70.29 (50.05–98.66)	20.82 (17.45–28.23)	107.01 (64.79–104.82)	17.49 (14.24–421.48)	64.94 (52.87–79.76)	20.59 (16.22–26.13)	76.45 (60.23–97.02)
**7**	>25	>84.07	>25	>84.07	14.28 (9.40–21.70)	53.10 (39.38–71.63)	>25	>84.07
**8**	>25	>79.78	20.27 (15.94–25.77)	64.65 (50.84–82.20)	15.79 (11.71–21.30)	70.27 (63.44–92.69)	16.69 (13.18–21.13)	53.23 (42.04–67.40)
**9**	>25	>78.67	>25	>78.67	15.58 (9.26–26.20)	49.02 (29.14–82.44)	>25	>78.67
**10**	>25	>78.85	>25	>78.85	12.51 (7.41–18.12)	36.45 (21.59–52.80)	>25	>78.85
**11**	>25	>76.42	>25	>76.42	18.79 (11.29–26.25)	57.44 (34.51–80.24)	>25	>76.42
**DOX**	0.02 (0.01–0.44)	0.04 (0.02–0.81)	0.01 (0.01–0.06)	0.02 (0.02–0.11)	0.03 (0.01–1.07)	0.06 (0.02–2.00)	1.00 (0.44–2.01)	1.84 (0.81–3.70)

The table shows the IC_50_ values (mean inhibitory concentration) and the respective 95% confidence interval. HepG2 cells: human hepatocellular carcinoma; HCT116: human colon carcinoma; HL-60: human promyelocytic leukemia; MRC5: human lung fibroblast. Doxorubicin (DOX) was used as a positive control.

**Table 2:** Results of the MIC, CFM, and the ratio of the two concentrations to the amides derived from ferulic acid against fungi of the genus *Candida*.

Amides	*C. albicans* CBS 562					*C. krusei* CBS 573					*C. tropicalis* CBS 94				
	MIC		MFC		MIC/MFC reason^∗^	MIC		MFC		MIC/MFC reason^∗^	MIC		MFC		MIC/MFC reason^∗^
	*μ*g/mL	*μ*mol/mL	*μ*g/mL	*μ*mol/mL		*μ*g/mL	*μ*mol/mL	*μ*g/mL	*μ*mol/mL		*μ*g/mL	*μ*mol/mL	*μ*g/mL	*μ*mol/mL	
2	N.A.	N.A.	N.A.	N.A.	—	N.A.	N.A.	N.A.	N.A.	—	N.A.	N.A.	N.A.	N.A.	—
3	N.A.	N.A.	N.A.	N.A.	—	N.A.	N.A.	N.A.	N.A.	—	N.A.	N.A.	N.A.	N.A.	—
4	N.A.	N.A.	N.A.	N.A.	—	N.A.	N.A.	N.A.	N.A.	—	N.A.	N.A.	N.A.	N.A.	—
5	N.A.	N.A.	N.A.	N.A.	—	N.A.	N.A.	N.A.	N.A.	—	N.A.	N.A.	N.A.	N.A.	—
6	500	1.85	500	1.85	1	250	0.82	250	0.82	1	N.A.	N.A.	N.A.	N.A.	—
7	N.A.	N.A.	N.A.	N.A.	—	500	1.68	500	1.68	1	500	1.68	500	1.68	1
8	500	1.59	500	1.59	1	500	1.59	500	1.59	1	500	1.59	500	1.59	1
9	N.A.	N.A.	N.A.	N.A.	—	500	1.57	500	1.57	1	500	1.57	500	1.57	1
10	62.5	0.18	62.5	0.18	1	125	0.18	125	0.18	1	500	1.45	500	1.45	1
11	62.5	0.19	125	0.38	2	62.5	0.19	62.5	0.19	1	500	1.52	500	1.52	1
Nystatin	3.75	0.0043	3.75	0.0043	1	3.75	0.0043	3.75	0.0043	1	3.75	0.0043	3.75	0.0043	1
Control medium	—	—	—	—	—	—	—	—	—	—	—	—	—	—	—
Fungal growth control	+	+	+	+	+	+	+	+	+	+	+	+	+	+	+

^∗^CFM/CIM reason ≥ 4 fungistatic or <4 fungicide; N.A.: no activity 2.4. Mechanism of fungicidal action.

**Table 3:** Results of the mechanisms of action: effects of compounds **10** and **11** in the presence or absence of a protective osmotic (sorbitol 0.8 M), as well as in the presence or absence of ergosterol.

Compounds	With ergosterol	Without ergosterol	With sorbitol	Without sorbitol
10	62.25 *μ*g/mL	62.25 *μ*g/mL	>1000 *μ*g/mL	31.25 *μ*g/mL
11	62.25 *μ*g/mL	62.25 *μ*g/mL	>1000 *μ*g/mL	15.62 *μ*g/mL
Caspofungin	—	—	1 *μ*g/mL	0.015 *μ*g/mL
Nystatin	31.25 *μ*g/mL	3.75 *μ*g/mL	—	—

**Table 4:** Details of the structural models of the investigated proteins.

UniProt accession	ID^(a)^	Description	Structure source^(b)^	PDB template	Coverage^(c)^	QMEAN^(d)^
A0A1D8PKB6	KRE6	Beta-glucan synthesis-associated protein KRE6	AlphaFold	N.A^**(e)**^	N.A	N.A
P29717	XOG1	Glucan 1,3-beta-glucosidase	PDB, 4 m82	N.A	N.A	N.A
A0A1D8PFV8	BHSD	17-Beta-hydroxysteroid dehydrogenase	AlphaFold	N.A	N.A	N.A
A0A1D8PMH9	CWH41	Mannosyl-oligosaccharide glucosidase	SwissModel	4j5t	92%	-2.90
Q5AEC0	SKN2	Involved in beta-1,6 glucan biosynthesis	AlphaFold	N.A	N.A	N.A
A0A1D8PTY8	BGL22	Beta-glucosidase	SwissModel	5fji	90%	-2.00
P43076	PHR1	pH-responsive protein 1	SwissModel	5oa2	84%	-2.04
A0A1D8PKY4	PHR3	1,3-Beta-glucanosyltransferase	SwissModel	5fih	64%	-1.70
Q5AJY5	PGA4	1,3-Beta-glucanosyltransferase	SwissModel	5oa2	73%	-1.64
Q59VW6	PGA5	1,3-Beta-glucanosyltransferase	SwissModel	5oa6	66%	-1.95
O42825	RHO1	GTP-binding protein RHO1	SwissModel	6sge	90%	0.76
P46598	HSP90	Heat shock protein 90	PDB, 6cjj	N.A	N.A	N.A
Q5ANK2	PKC1	Protein kinase C	SwissModel	4otd	30%	-1.29
Q5AAG6	MKC1	Mitogen-activated protein kinase MKC1	SwissModel	5z33	70%	-1.29
A0A1D8PR87	BCK1	Mitogen-activated protein kinase kinase kinase	SwissModel	2xik	20%	-1.57
Q5AAU5	ATC1	Cell wall acid trehalase ATC1	AlphaFold	N.A	N.A	N.A
Q873N2	GWT1	GPI-anchored wall transfer protein 1	AlphaFold	N.A	N.A	N.A
Q5AGA0	MNN1	Alpha-1,3-mannosyltransferase MNN1	AlphaFold	N.A	N.A	N.A
A0A1D8PCT0	GSC1	1,3-Beta-D-glucan-UDP glucosyltransferase	AlphaFold	N.A	N.A	N.A

^(a)^ID of each target along the manuscript. ^(b)^Source of the structural model: Protein Data Bank (PDB), homology model (SwissModel), or AlphaFold repository (AlphaFold). ^(c)^Coverage of the query sequence by the template. ^(d)^Swiss-Model QMEAN4 score. ^(e)^Not applicable.

**Table 5:** Summary of the docking of compounds 10 and 11 to their potential targets.

Compound	Target	Pose	CHEMPLP		GoldScore		ChemScore		ASP		Consensus *Z*-score
			Score	*Z*-score	Score	*Z*-score	Score	*Z*-score	Score	*Z*-score	
10	KRE6	1	76.15	2.14	5.87	0.74	24.01	1.49	44.78	1.43	1.45
	XOG1	1	55.52	1.29	10.06	0.39	13.89	1.71	38.65	2.74	1.53
	BHSD	1	73.4	2.02	20.21	0.34	28.65	1.91	54.73	2.38	1.66
	CWH41	1	57.23	1.86	4.6	0.61	24.55	2.05	38.74	1.63	1.54
	SKN2	1	78.99	2.08	30.65	0.96	25.08	0.84	49.72	1.68	1.39
	BGL22	1	46.08	0.51	8.4	0.75	18.7	1.28	30.21	1.28	0.96
	PHR1	1	65.57	2.24	16.01	-0.01	23.22	2.1	40.92	1.89	1.55
	PHR3	1	57.21	2.49	-8.43	-0.21	18.41	2.08	27.83	1.39	1.44
	PGA4	1	59.35	2.57	-4.46	-0.87	17.26	2.58	31	1.25	1.38
	PGA5	1	65.48	2.29	31.49	0.88	20.7	1.92	36.38	1.42	1.63
	RHO1	1	73.79	1.32	41.69	1.07	28.65	1.82	31.37	1.77	1.5
	HSP90	1	67.98	2.58	24.02	1.18	27.93	2.51	28.26	1.55	1.96
	PKC1	1	68.13	2.05	3.82	0.53	24.15	1.4	29.29	0.76	1.18
	MKC1	1	69.33	1.95	26.93	1.04	25.16	1.51	33.19	2.47	1.74
	BCK1	1	60.11	1.42	24.84	0.78	22.65	1.41	26.34	2.65	1.57
	ATC1	1	53.87	1.71	12.25	0.3	18.67	1.91	37.84	1.59	1.38
	GWT1	1	79.91	3.35	26.6	0.53	22.7	-0.26	38.93	1.78	1.35
	MNN1	1	55.61	0.61	26.52	0.71	19.83	1.16	31.37	0.9	0.84
	GSC1	1	62.94	1.22	23.42	0.23	28.7	2.79	39.7	2	1.56
11	KRE6	1	62.99	1.19	34.72	1.24	22.3	1.72	48.95	2.26	1.6
	XOG1	1	57.23	1.81	37.54	1.26	13.03	1.15	31.6	1.11	1.33
	BHSD	1	78.28	2.29	35.72	0.8	30.08	2.21	60.91	2.93	2.05
	CWH41	1	60.76	1.94	17.63	0.38	17.89	0.35	35.64	0.96	0.91
	SKN2	1	80.13	2.88	45.86	1.55	32.61	2.3	54.6	2.04	2.19
	BGL22	1	45.34	1.29	-6.76	-0.24	17.98	1.84	32.38	2.23	1.28
	PHR1	1	63.75	1.65	27.76	0.46	24.32	2.43	42.4	1.8	1.58
	PHR3	1	48.61	1.07	25.56	1.03	15.96	1.9	32.82	1.71	1.43
	PGA4	1	58.91	2.04	40.73	1.54	12.04	1.14	29.06	0.85	1.39
	PGA5	1	61.9	1.24	48.19	1.95	16.88	0.74	35.36	0.91	1.21
	RHO1	1	76.79	1.38	56.2	1.65	30.2	2.52	32.16	1.64	1.8
	HSP90	1	61.14	1.67	9.44	-0.43	24.03	1.55	27.17	1.14	0.98
	PKC1	1	63.7	1.74	18.62	1.24	20.99	0.61	31.15	1.35	1.24
	MKC1	1	66.27	1.13	43.14	2	23.97	1.47	29.66	1.22	1.45
	BCK1	1	59.88	2.27	22.94	0.47	21.73	1.64	25.72	2.53	1.73
	ATC1	1	55.21	1.75	12.38	0.31	10.48	0.82	38.66	1.47	1.09
	GWT1	1	82.79	3.65	41.5	0.99	25.65	1.93	38.01	1.52	2.02
	MNN1	1	59.9	1.81	32.55	0.92	19.8	1.41	31.06	0.88	1.26
	GSC1	1	63.71	1.62	31.36	0.57	22.29	1.31	42.63	2.34	1.46

## Data Availability

The data presented in this study are available in the article's Supplementary Materials.

## Supplementary Materials

Supplementary Materials Table S1: full results of molecular docking calculations. Table S2: free energies of binding estimated from MD calculations. Table S3: reference ligands and residues for defining the docking binding cavities. Figures S33 and S34: plots of RMSD vs. MD snapshot for the the predicted complexes of compounds 10 and 11 with GWT1 and GSC1.
