# Supplementary material for: Cytotoxic and Antifungal Amides Derived from Ferulic Acid: Molecular Docking and Mechanism of Action
Source: Biomed Res Int. 2021 Nov 1;2021:3598000. doi: 10.1155/2021/3598000 (PMC8575619; doi:10.1155/2021/3598000)
Supplement: Supplementary Materials — Table S1: full results of molecular docking calculations. Table S2: free energies of binding estimated from MD calculations. Table S3: reference ligands and residues for defining the docking binding cavities. Figures S33 and S34: plots of RMSD vs. MD snapshot for the the predicted complexes of compounds 10 and 11 with GWT1 and GSC1. [file 3598000.f1.docx]

Supplementary materials

Cytotoxic and Antifungal Amides Derived from Ferulic Acid: Molecular Docking and Mechanism of Action

Mayara Castro de Morais^1^, Yunierkis Perez-Castillo^2^, Valdenizia Rodrigues Silva^3^, Luciano de Souza Santos^3^, Milena Botelho Pereira Soares^3^, Daniel Pereira Bezerra^3^, Ricardo Dias de Castro^4^, and Damião Pergentino de Sousa^1^*.

^1^ Laboratory of Pharmaceutical Chemistry, Department of Pharmaceutical Sciences, Federal University of Paraíba, 58051-900 João Pessoa, PB, Brazil. E-mail: mayaracastrodemorais@gmail.com

^2^ Escuela de Ciencias Físicas y Matemáticas, Universidad de Las Américas, Quito, Ecuador. E-mail: yunierkis.perez@udla.edu.ec

^3^ Instituto Gonçalo Moniz, Fundação Oswaldo Cruz (IGM-FIOCRUZ/BA), Salvador 40296-710, Bahia, Brazil. E-mails: VRS: [valdeniziar@gmail.com](mailto:valdeniziar@gmail.com); LSS: [luciano.biomed@gmail.com](mailto:luciano.biomed@gmail.com); MBPS: [milena.soares@fiocruz.br](mailto:milena.soares@fiocruz.br); DPB: danielpbezerra@gmail.com

^4^ Laboratory of Experimental Pharmacology and Cell Culture, Department of Clinical and Social Dentistry, Federal University of Paraíba, 58051-900 Joao Pessoa, PB, Brazil. E-mail: rcastro@ccs.ufpb.br

***** Correspondence: damiao_desousa@yahoo.com.br; Tel.: +55-83-3216-7347.

**Abstract:** Amides derived from ferulic acid have a wide spectrum of pharmacological activities, including antitumor and antifungal activity. In the present study, a series of ten amides were obtained by coupling reactions using the reagents PyBOP ((Benzotriazol-1-yloxy) tripyrrolidinophosphoniumhexafluorophosphate) and DCC (N,N′-dicyclohexylcarbodiimide). All the compounds were identified on the basis of their IR, ^1^H and ^13^C NMR, HRMS data, and with yields ranging from 43.17 to 91.37%. The compounds were subjected to cytotoxic tests by the alamar blue technique and antifungal screening by the broth microdilution method to determine the minimum inhibitory concentration (MIC). The amides **10** and **11** had the best result in both biological evaluations, and compound **10** was the most potent and selective in HL-60 cancer cells, with no cytotoxicity in healthy cells. This amide had antifungal activity in all strains and had the lowest MIC against *Candida albicans* and *Candida tropicalis*. The possible mechanism of antifungal action occurs via the fungal cell wall. Molecular modeling suggested that compounds **10** and **11** interact with the enzymes GWT1 and GSC1, which are essential for the development of *C. albicans*. The findings of the present study demonstrated that compounds **10** and **11** may be used as a platform in drug development in the future.

**Keywords:** cinnamic acids; natural products; fungi; cancer; antitumor; *Candida.*

1. Experimental
   1. *Chemical Characterization Compounds 2-11*

*(E)-3-(4-hydroxy-3-methoxyphenyl)-N-isobutylacrylamide (****2****)*

White amorphous solid; yield 67% (0.08518 g, 0.3416 mmols); m.p.: 75-81°C; TLC (6:4 hexane/EtOAc), Rf = 0.44; IRν_max_ (KBr, cm^−1^) 3286, 3083, 2961-2934, 1656, 1592, 1548 and 1516; ^1^H NMR (200 MHz, CdCl_3_) δ_H_ 7.84 (*d*, J=15.5 Hz, H-7, H=1), 7.34- 7.15 (*m*, H-2, H-5, H-6, H=3), 6.64 (*d*, J=15.5 HZ, H-8, H=1); 6.45 (*s*, N-H, H=1); 4.15 (*s*, 3H, OCH_3_); 3.51 (*t*, J= 6.6 Hz, H-1’, H=2); 2.13 (*m*, H-2’, H=1); 0.94 (*d*, H-3’, J=6.6 Hz, H=6) ; ^13^C NMR (50 MHz, CdCl_3_) 166.61 (C=O), 147.48 (C-3), 146.85 (C-4), 141.08 (C-7), 127.23 (C-1), 121.95 (C-6), 118.06 (C-8), 114.83 (C-5), 109.71 (C-2), 55.74 (C-9), 47.13 (C-1’), 28.64 (C-2’), 20.11 (C-3’); HRMS (MALDI): calculated for C_14_H_19_NO_3_ [M+H]^+^ : 250.140; found 250.191.

*(E)-3-(3-hydroxy-4-methoxyphenyl)-1-(pyrrolidin-1-yl)prop-2-en-1-one (****3****)*

Yellow amorphous solid; yield 83% (0.08755g, 0.335 mmols); m.p.: 158-162°C (lit. 160°C ^1^); TLC (6:4 hexane/EtOAc), Rf = 0.42; IR ν_max_ (KBr, cm^−1^) 3108, 3012, 2979-2892, 1657, 1618, 1579 and 1532. ^1^H NMR (200 MHz, CdCl_3_) δ_H_ 7.64 (*d*, H-7, J=15.5 Hz, H=1), 7.11- 6.89 (*m*, H-2, H-5, H-6, H=3), 6.56 (*d*, J=15.5 HZ, H-8, H=1); 3.91 (*s*, 3H), 3.61 (*s*, 4H), 1.94 (*s*, 4H); ^13^C NMR (50 MHz, CdCl_3_) 165.28, 147.59, 146.88, 127.82, 121.93, 116.12, 114.91, 110.35, 110.20, 56.16,46.49, 39.88 ^2^.

*(E)-N-cyclohexyl-3-(3-hydroxy-4-methoxyphenyl)acrylamide (****4****)*

Amber amorphous solid; yield 62% (0.08755g, 0.31 mmols); m.p.: 160-165°C (lit. 160°C ^1^); TLC (6:4 hexane/EtOAc), Rf = 0.40; IR ʋmax (KBr, cm^−1^): 3283, 3066, 2936-2855, 1654, 1617, 1542 and 1514. ^1^H NMR (200 MHz, CdCl_3_) δ_H_ 7.53 (*d*, H-7, J=15.5 Hz, H=1), 7.05-6.86 (*m*, H-2, H-5, H-6, H=3), 6.26 (*d*, J=15.6 Hz, H-8, H=1), 5.69 (*sl*, O-H, H=1), 3.89 (*s*, 3H, OCH_3_), 1.53-0.84 (*m*, H-2, H-5, H-6, H=3); ^13^C NMR (50 MHz, CdCl_3_) 165.46, 147.45, 146.82, 141.13, 127.49, 122.27, 118.48, 114.82, 109.75, 56.08, 48.67, 33.37, 25.66, 24.99 ^2^.

*(E)-3-(3-hydroxy-4-methoxyphenyl)-N-phenylacrylamide (****5****)*

Yellow amorphous solid; yield 90% (0.13202 g, 0.47 mmols); m.p.: 148-153°C (lit. 152-154°C ^2^); TLC (6:4 hexane/EtOAc), Rf = 0.40; IR ν_max_ (KBr, cm^−1^) 3361, 3065, 2944, 1654, 1583 ,1555 and 1518. ^1^H NMR (200 MHz, CdCl_3_) δ_H_ 9.88 (*s*, NH, H=1), 7.45 (*d*, *J* = 8.0 Hz, H-2’, H=2), 7.26 (*d*, *J* = 15.6 Hz, H-7, H=1), 7.06 (*t*, *J* = 7.7 Hz, H-3’, H=2), 6.93 (*s*, H-2, H=1); 6.84 – 6.74 (*m*, H-5, H-6, H=2), 6.58 (*d*, *J* = 8.1 Hz, H-4’, H=1), 6.41 (*d*, *J* = 15.6 Hz, H-8, H=1), 3.57 (*s*, OCH_3_, H=3); ^13^C NMR (50 MHz, CdCl_3_) 166.61, 147.67, 146.95, 141.58, 138.24, 128.64, 127.16, 122.17, 118.08, 114.95, 109.90, 105.03, 105.01, 43.82 ^2^.

*(E)-N-benzyl-3-(3-hydroxy-4-methoxyphenyl)acrylamide (****6****)*

Amber amorphous solid; yield 70% (0.09845g, 0.3655 mmols); 148-152°C (lit. 155 °C ^2^); TLC (6:4 hexane/EtOAc), Rf = 0.36; IR ν_max_ (KBr, cm^−1^) 3337, 3090, 2977, 1656, 1621, 1587 and 1552. ^1^H NMR (200 MHz, CdCl_3_) δ_H_ 7.85 (*d*, H-7, J=15.5 Hz, H=1), 7.56 (*s*, H-2’, H-3’, H-4’, H-5’ H=5), 7.28-7.11 (*m*, H-2, H-5, H-6, H=3), 6.90 (*s*, N-H, H=1), 6.64 (*d*, H-8, J=15.6 Hz, H=1), 4.79 (*d*, H-1’, J=4.8 Hz, H=1), 4.07 (*s*, 3H, OCH_3_). ; ^13^C NMR (50 MHz, CdCl_3_) 166.62, 147.67, 146.98, 141.62, 138.24, 128.63, 127.71, 122.13, 122.03, 117.97, 117.79, 114.95, 109.91, 55.94, 43.81 ^2^.

*(E)-3-(3-hydroxy-4-methoxyphenyl)-N-(4-methylbenzyl)acrylamide (****7****)*

White amorphous solid; yield 65% (0.09895g, 0.3327 mmols); m.p.: 147-150°C (lit. 143°C ^3^); TLC (6:4 hexane/EtOAc), Rf= 0.33; IR ν_max_ (KBr, cm^−1^) 3327, 3288, 2938- 2838, 1655, 1592 and 1427. ^1^H NMR (200 MHz, DMSO-d6) δ_H_ δ 8.28 (*s*, N-H, H=1), 7.19 (*d*, *J* = 15.7 Hz, H-7, H=1), 6.95 (*sl*, H-6, H-3’, H-4’, H=5 ), 6.82 (*d*, *J* = 8.1 Hz, H-2, H=1), 6.61 (*d*, *J* = 8.1 Hz, H-5, H=1), 6.33 (*d*, *J* = 15.7 Hz, H-8, H=1), 4.15 (*d*, *J* = 5.3 Hz, H-1’, H=2), 3.61 (*s*, OCH_3_, H=3), 2.07 (*s*, CH_3_, H=3); ^13^C NMR (50 MHz, DMSO-d6) 165.60, 148.43, 147.96, 139.70, 136.60, 136.06, 129.01, 127.50, 126.51, 121.75, 118.80, 115.67, 110.85, 55.47, 42.18, 23.68 ^4^.

*(E)-3-(3-hydroxy-4-methoxyphenyl)-N-(4-methoxybenzyl)acrylamide (****8****)*

White amorphous solid; yield 43% (0.069 g, 0.2320 mmols); m.p.: 134-139°C; TLC (6:4 hexane/EtOAc), Rf = 0.29; IR ν_max_ (KBr, cm^−1^) 3356, 3079, 2957- 2839, 1654, 1592 and 1454. ^1^H NMR (200 MHz, CdCl_3_) δ_H_ 7.87 (*d*, *J* = 15.6 Hz, H-7, H=1), 7.56-7.51 (*m*, H-2, H-6, H=2), 7.34-7.12 (*m*, H-5, H-3’, H-4’, H=5), 6.59 (*d*, *J* = 15.5 Hz, H-8, H=1), 6.47 (*s*, N-H, H=1), 4.77 (*d*, *J* = 4.8 Hz, H-1’, H=2), 4.15 (*s*, OCH_3_, H=3), 4.09 (*s*, OCH_3_, H=3); ^13^C NMR (50 MHz, CdCl_3_) 166.32 (C=O), 147.60 (C-5’), 159.11 (C-4), 146.87 (C-3), 141.58 (C-7), 130.34 (C-2’), 129.46 (C-3’), 127.31 (C-1), 122.20 (C-6), 117.82 (C-8), 114.87 (C-2), 114.16 (C-4’), 109.85 (C-5), 55.90 (C-6’), 55.48 (C-9), 43.46 (C-1’); HRMS (MALDI) calculated for C_18_H_19_NO_4_ [M+H]^+^: 314.139; found 314.144.

*(E)-N-(4-chlorobenzyl)-3-(3-hydroxy-4-methoxyphenyl)acrylamide (****9****)*

Amber amorphous solid; yield 47% (0.07597g, 0.2390 mmols); m.p.: 128-130°C (lit. 129-131°C ^4^); TLC (6:4 hexane/EtOAc), Rf = 0.26; IR ν_max_ (KBr, cm^−1^) 3335, 3086, 2971, 1657, 1590, 1559 and 1514; ^1^H NMR (200 MHz, CdCl_3_) δ 7.47 (*d*, *J* = 15.5 Hz, H-7, H=1), 7.18-7.08 (*m*, H-3’, H-4’, H=4), 6.96-6.74 (*m*, 2-H, 5-H, 6-H, H=3), 6.41 (*s*, N-H, H=1), 6.24 (*d*, *J* = 15.6 Hz, H-8, H=1), 4.38 (*d*, *J* = 5.8 Hz, H-1’, H=2), 3.73 (*s*, OCH_3_, H=3); ^13^C NMR (50 MHz, CdCl_3_) 166.46, 147.54, 146.76, 141.65, 136.90, 133.26, 129.16, 127.16, 122.22, 117.89, 114.94, 109.98, 109.87, 55.83, 43.11 ^5^.

*(E)-N-(3,4-dimethoxybenzyl)-3-(3-hydroxy-4-methoxyphenyl)acrylamide (****10****)*

Yellow amorphous solid; yield 52% (0.09193g, 0.2678 mmols); m.p.: 65-70°C (lit. 63.7−65.9°C ^5^); TLC (6:4 hexane/EtOAc), Rf = 0.15; IR ν_max_ (KBr, cm^−1^) 3227, 3063, 2937, 1652, 1593**,** 1549 and 1515; ^1^H NMR (200 MHz, CdCl_3_) δ_H_ δ 7.53 (*d*, *J* = 15.5 Hz, H-7, H=1), 6.98 – 6.70 (*m*, H-2, H-5, H-6, H-3’, H-6’, H-7’, H=6), 6.28 (*d*, H-8, J=15.6 Hz, H=1), 5.25 (*s*, OH, H=1), 4.42 (*d*, *J* = 5.6 Hz, H-1’, H=2), 3.79 (*s*, OCH_3_, H=9). ^13^C NMR (50 MHz, CdCl_3_) 166.39, 149.12, 148.45, 147.64, 146.92, 141.50, 130.84, 127.22, 122.17, 122.09, 120.22, 117.84, 114.90, 111.21, 109.82, 55.94, 43.75 ^6^.

*(E)-N-(benzo[d][1,3]dioxol-5-ylmethyl)-3-(3-hydroxy-4-methoxyphenyl) acrylamide (****11****)*

Amber amorphous solid; yield 70% (0.11748g, 0.3591 mmols), MM: 327.11 g/mol, m.p.: 172-175 °C (lit. 170°C ^6^); TLC (6:4 hexane/EtOAc), Rf = 0.12; IR ν_max_ (KBr, cm^−1^) 3300, 3030, 2896, 1651, 1598, 1534 and 1506. ^1^H NMR (200 MHz, DMSO-d6: 8.26 (*s,* N-H, H=1), 7.19 (*d*, J= 15.7, H-7, H=1), 6.95 (*s*, H-2, H=1), 6.83 (*d*, J= 8.2, H-6, H=1), 6.89- 6.40 (*m*, H-5, H-3’, H-6’ H-7’, H=4), 6.32 (*d*, J= 15.7 Hz, H-8, H=1), 5.79 (*s*, H-8’, H=2 ), 4.11 (*d*, J= 5.7 Hz, H-1’, H=2), 3.62 (*s*, OCH_3_, H=3); ^13^C NMR (50 MHz, DMSO-d6) 165.54, 148.40, 147.92, 147.34, 143.16, 139.56, 133.53, 126.47, 121.71, 121.71, 120.65, 118.84, 115.75, 110.84, 108.12, 100.92, 55.58, 42.20 ^7^.

**
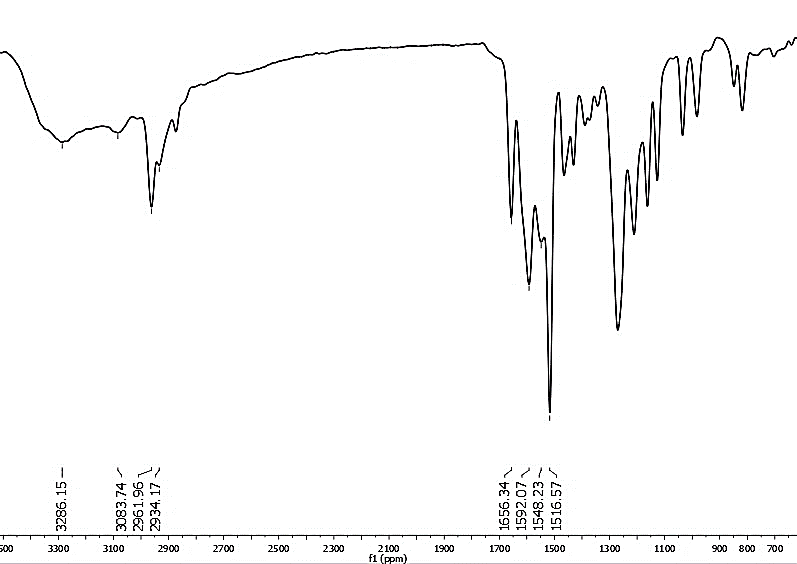
**

**Figure S1.** IR ʋmax (KBr, cm^-1^) spectrum of (*E*)-3-(4-hydroxy-3-methoxyphenyl)-*N*-isobutylacrylamide (**2**)

**
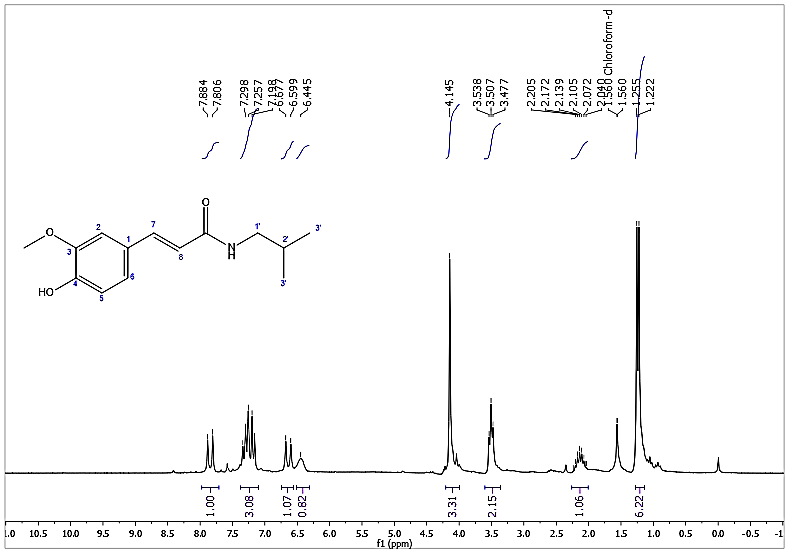
**

**Figure S2.** ^1^H NMR (CdCl_3_, 200 MHz) spectrum of (*E*)-3-(4-hydroxy-3-methoxyphenyl)-*N*-isobutylacrylami-de (**2**)


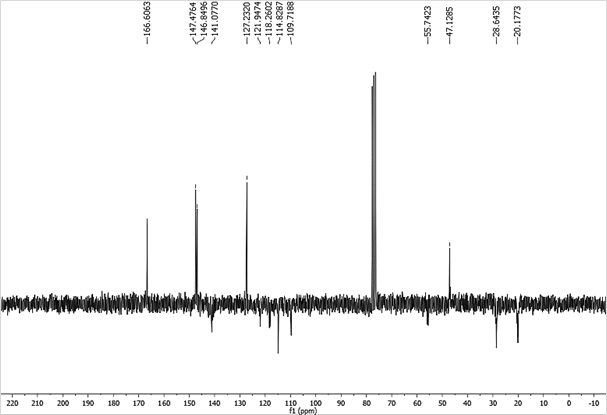


**Figure S3.** ^13^C NMR (CdCl_3_, 50 MHz) spectrum of (*E*)-3-(4-hydroxy-3-methoxyphenyl)-*N*-isobutylacrylami-de (**2**)

**
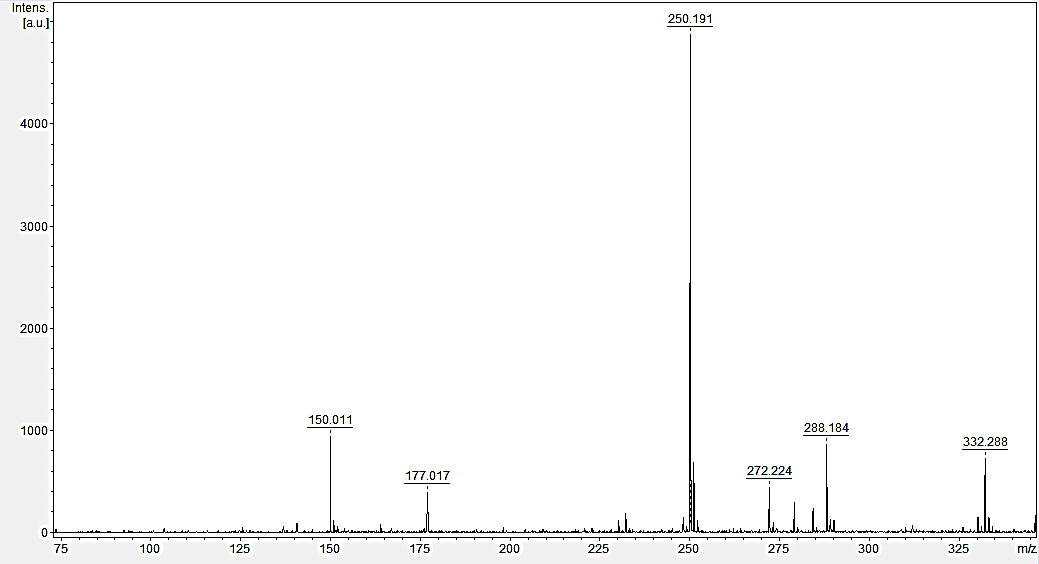
**

**Figure S4.** High resolution MALDI type mass spectrum of (*E*)-3-(4-hydroxy-3-methoxyphenyl) -N-isobutylacrylamide (**2**)


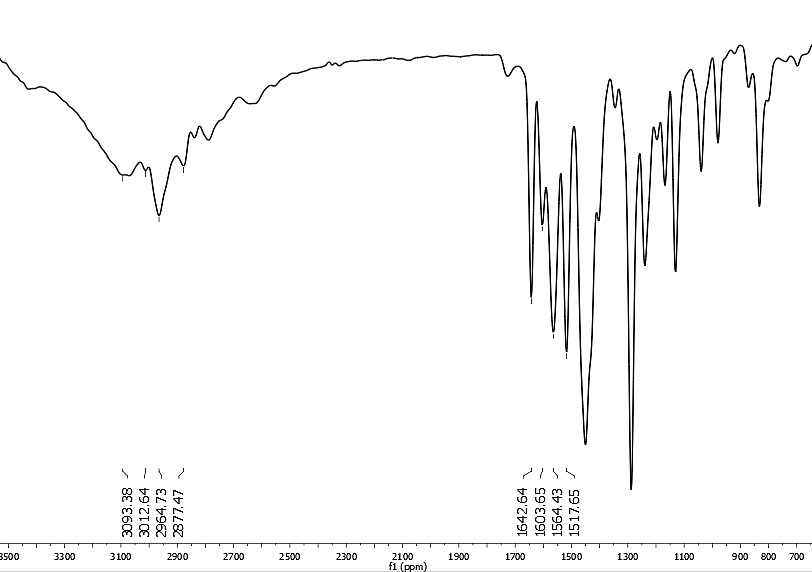


**Figure S5.** IR ʋmax (KBr, cm^-1^) spectrum of (*E*)-3-(3-hydroxy-4-methoxyphenyl)-1-(pyrrolidin-1-yl)prop-2-en-1-one (**3**)


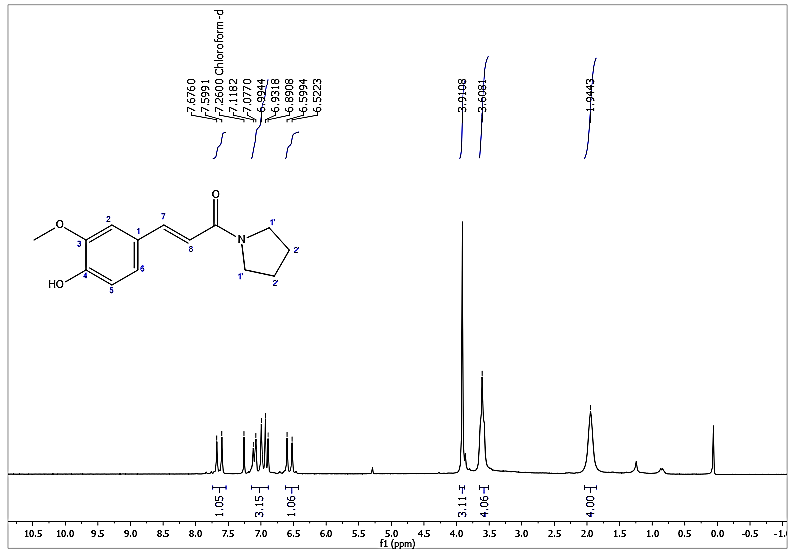
 **Figure S6**. ^1^H NMR (CdCl_3_, 200 MHz) spectrum of (*E*)-3-(3-hydroxy-4-methoxyphenyl)-1-(pyrrolidin-1-yl)prop-2-en-1-one (**3**)


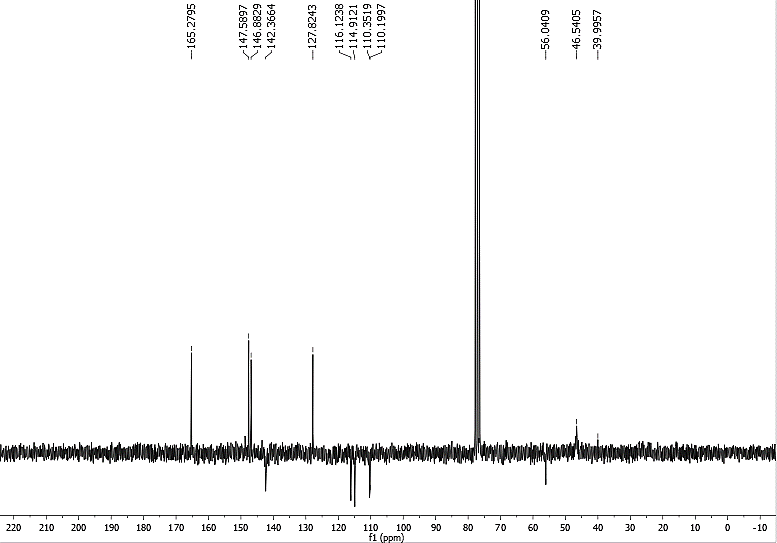


**Figure S7**. ^13^C NMR (CdCl_3_, 50 MHz) spectrum of (*E*)-3-(3-hydroxy-4-methoxyphenyl)-1-(pyrrolidin-1-yl)prop-2-en-1-one (**3**)


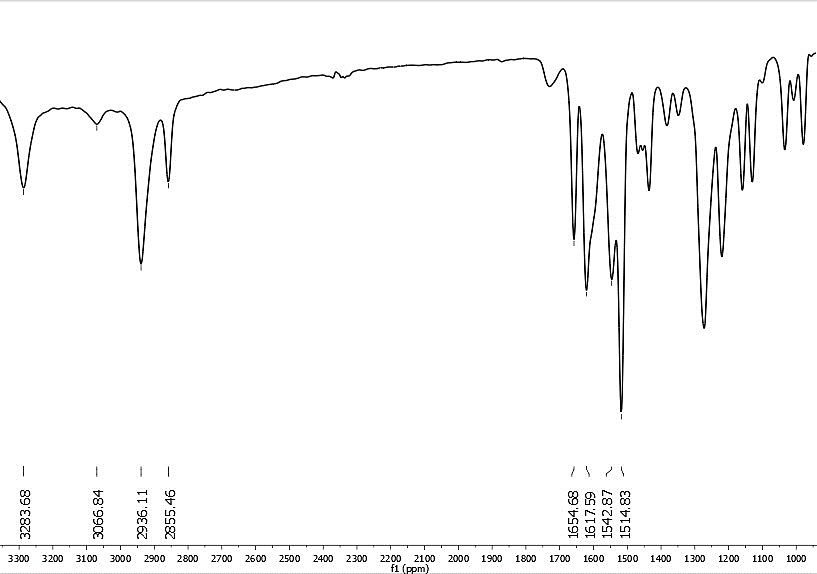


**Figure S8**. IR ʋmax (KBr, cm^-1^) spectrum of (*E*)-*N*-cyclohexyl-3-(3-hydroxy-4-methoxyphenyl)acrylamide (**4)**

**
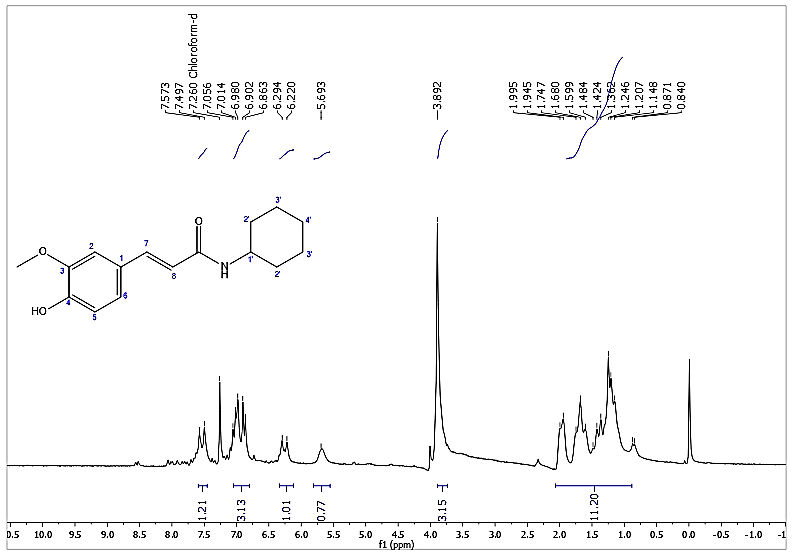
**

**Figure S9**. ^1^H NMR (CdCl_3_, 200 MHz) spectrum of (*E*)-*N*-cyclohexyl-3-(3-hydroxy-4-methoxyphenyl)acryla-mide (**4)**


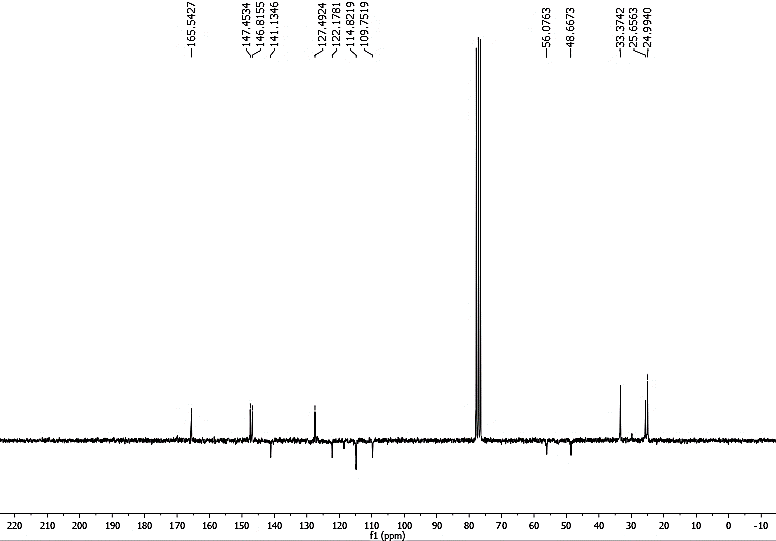


**Figure S10**. ^13^C NMR (CdCl_3_, 50 MHz) spectrum of (*E*)-*N*-cyclohexyl-3-(3-hydroxy-4-methoxyphenyl)acryla-mide (**4**)


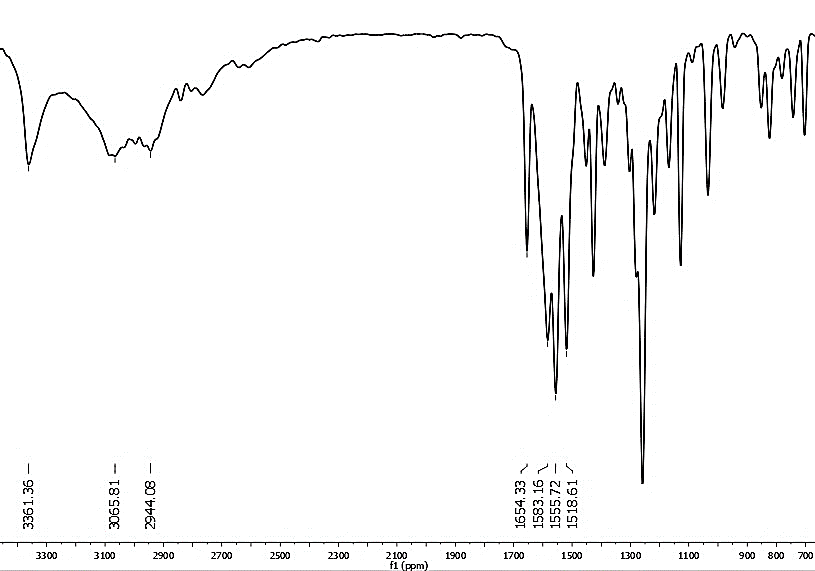


**Figure S11**. IR ʋmax (KBr, cm^-1^) spectrum of (*E*)-3-(3-hydroxy-4-methoxyphenyl)-*N*-phenylacrylamide (**5**)


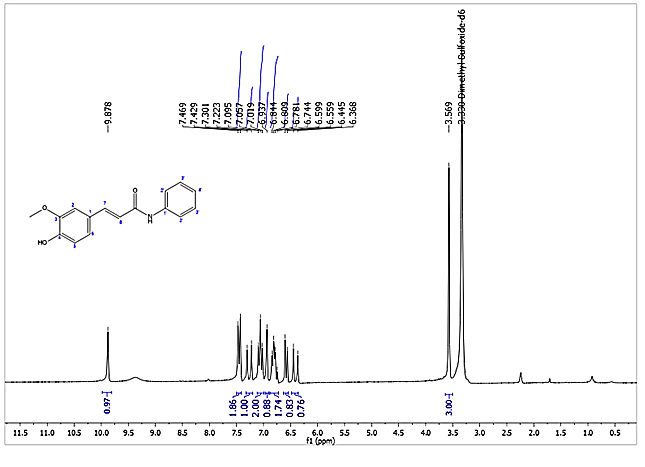
**Figure S12**. ^1^H NMR (CdCl_3_, 200 MHz) spectrum of (*E*)-3-(3-hydroxy-4-methoxyphenyl)-*N*-phenylacryla-mide (**5**)

**
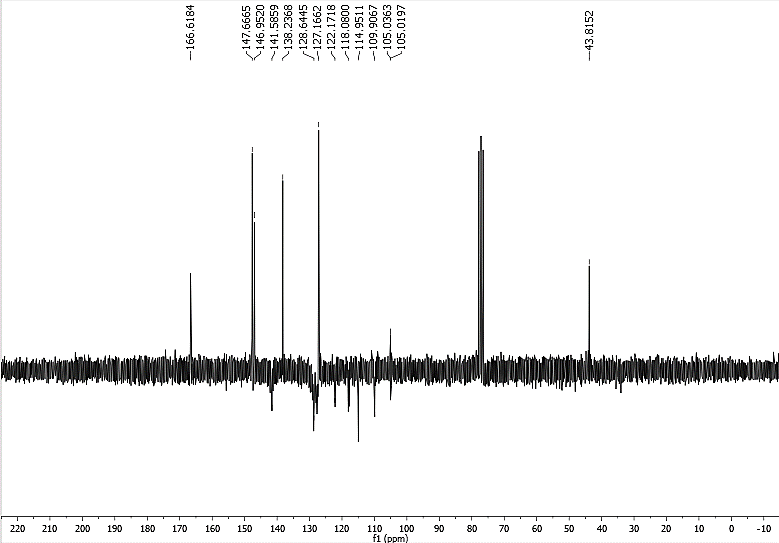
**

**Figure S13**. ^13^C NMR (CdCl_3_, 50 MHz) spectrum of (*E*)-3-(3-hydroxy-4-methoxyphenyl)-*N*-phenylacryla-mide (**5**)


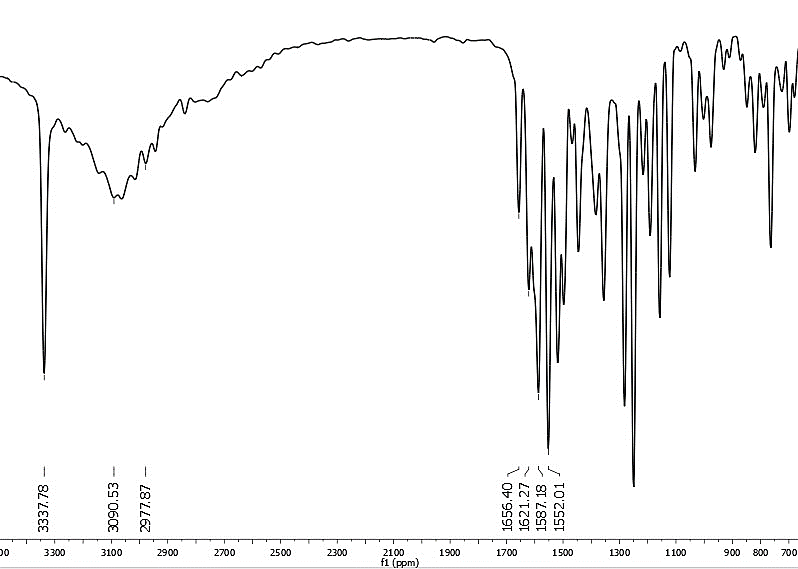


**Figure S14**. IR ʋmax (KBr, cm^-1^) spectrum of (*E*)-*N*-benzyl-3-(3-hydroxy-4-methoxyphenyl)acrylamide (**6**)


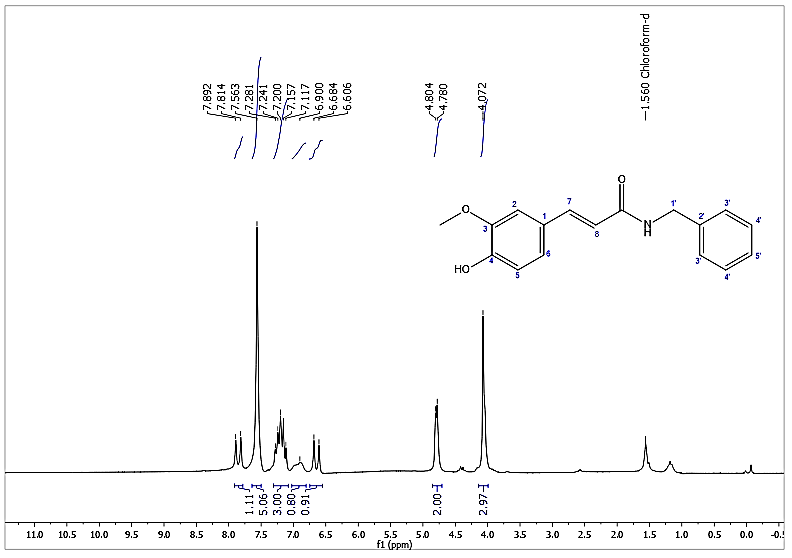


**Figure S15**. ^1^H NMR (CdCl_3_, 200 MHz) spectrum of (*E*)-*N*-benzyl-3-(3-hydroxy-4-methoxyphenyl)acryla-mide (**6**)


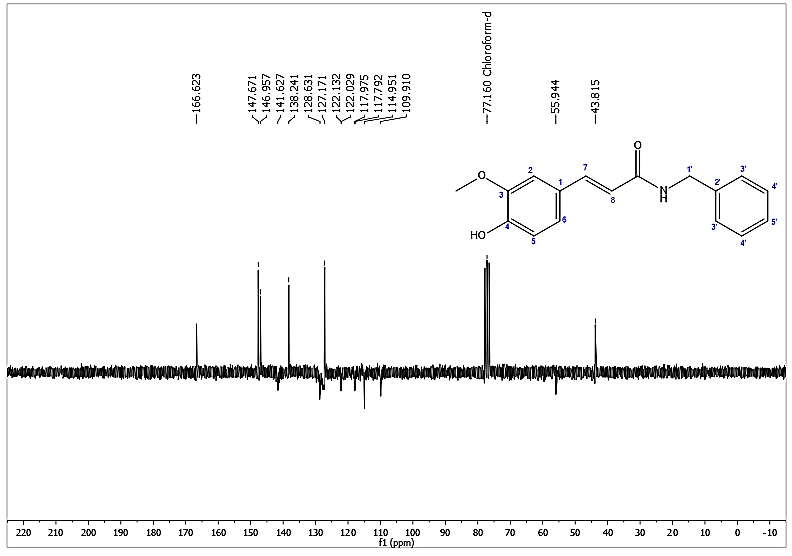


**Figure S16**. ^13^C NMR (CdCl_3_, 50 MHz) spectrum of (*E*)-*N*-benzyl-3-(3-hydroxy-4-methoxyphenyl)acryla-mide (**6**)


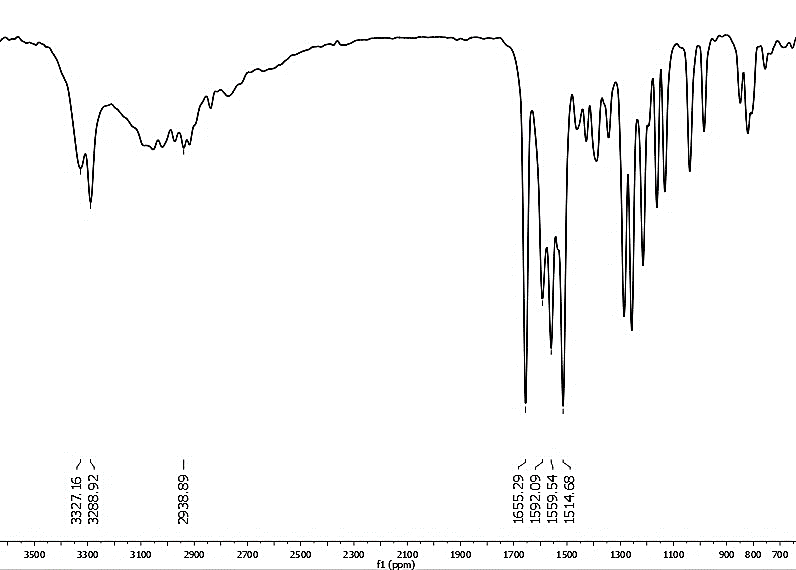


**Figure S17.** IR ʋmax (KBr, cm^-1^) spectrum of (*E*)-3-(3-hydroxy-4-methoxyphenyl)-*N*-(4-methylbenzyl)acryla-mide (**7**)

**
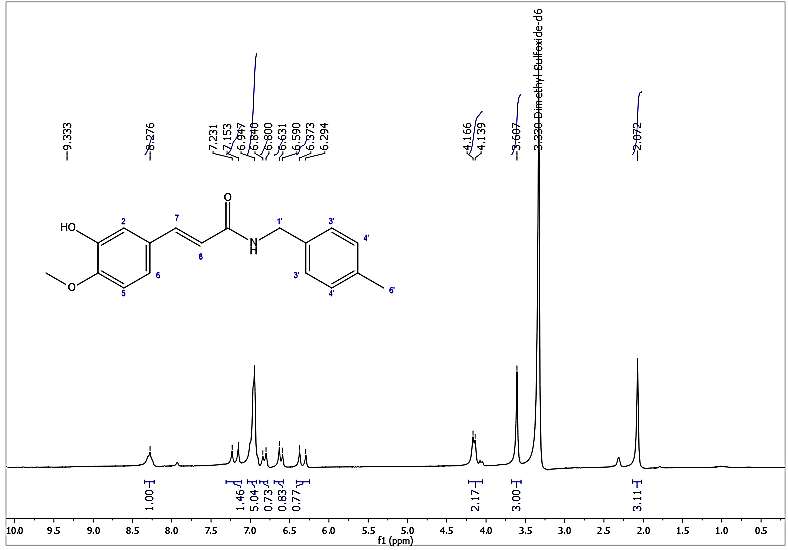
**

**Figure S18.** ^1^H NMR (DMSO-d6, 200 MHz) spectrum of (*E*)-3-(3-hydroxy-4-methoxyphenyl)-*N*-(4-methylbenzyl)acrylamide (**7**)


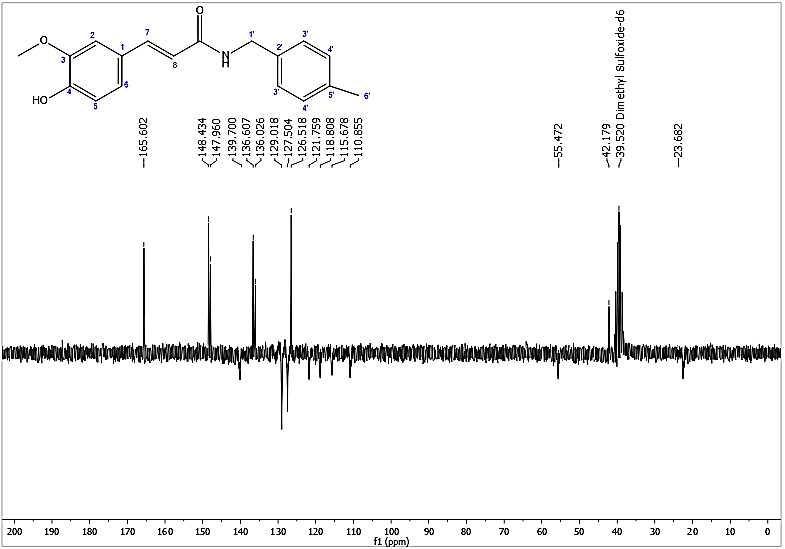


**Figure S19**. ^13^C NMR (DMSO-d6, 50 MHz) spectrum of (*E*)-3-(3-hydroxy-4-methoxyphenyl)-*N*-(4-methylbenzyl)acrylamide (**7**)


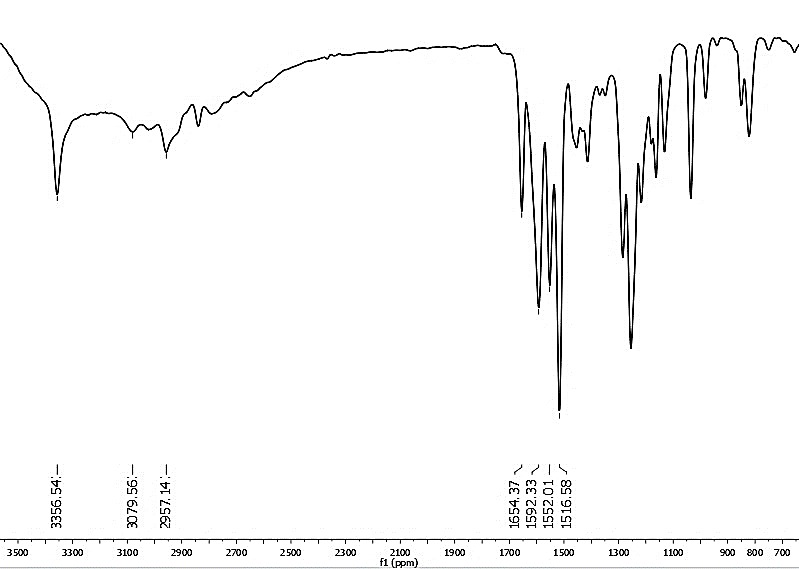


**Figure S20**. IR ʋmax (KBr, cm^-1^) spectrum of (*E*)-3-(3-hydroxy-4-methoxyphenyl)-N-(4-methoxybenzyl)acrylamide (**8**)

**
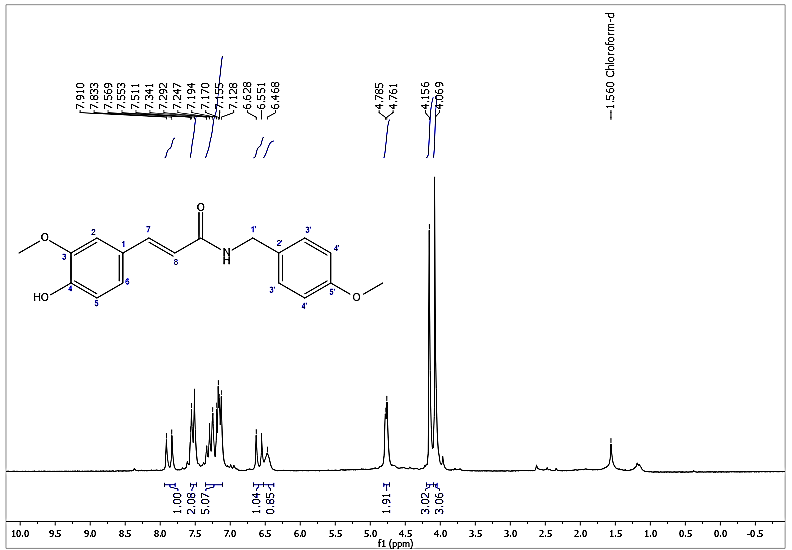
**

**Figure S21**. ^1^H NMR (CdCl_3_, 200 MHz) spectrum of (*E*)-3-(3-hydroxy-4-methoxyphenyl)-N-(4-methoxybenzyl)acrylamide (**8**)


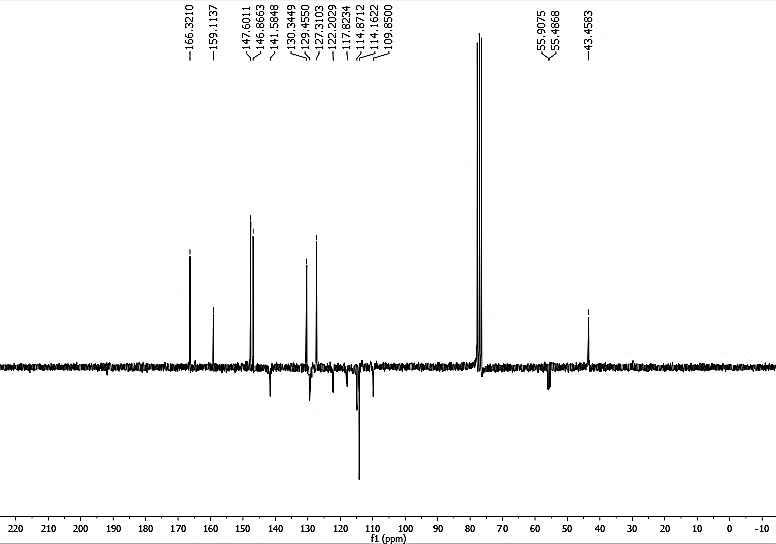


**Figure S22**. ^13^C NMR (CdCl_3_, 50 MHz) spectrum of (*E*)-3-(3-hydroxy-4-methoxyphenyl)-N-(4-methoxybenzyl)acrylamide (**8**)


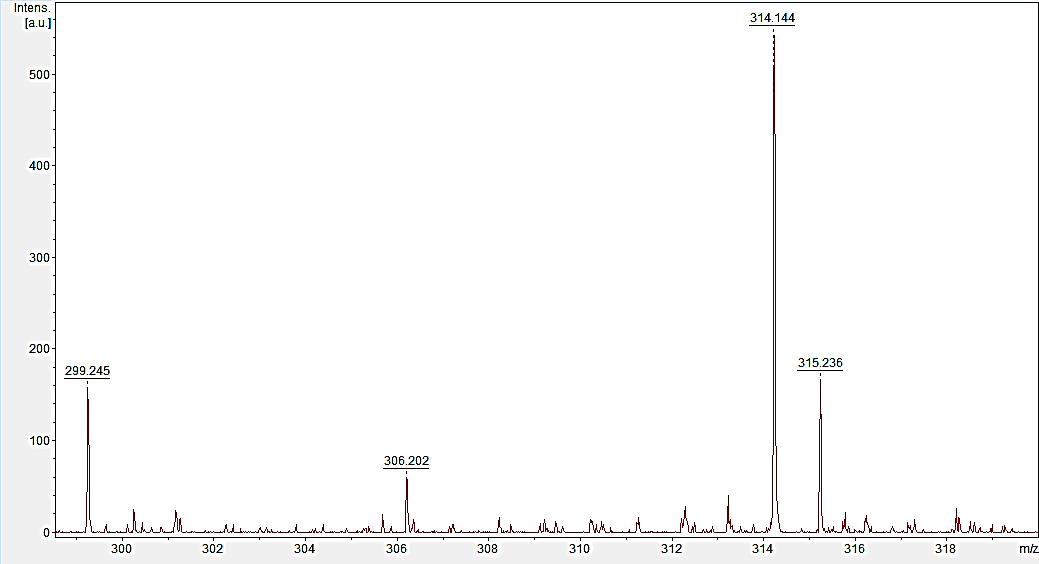


**Figure S23**. High resolution MALDI type mass spectrum of (E)-3-(3-hydroxy-4-methoxyphenyl)-N-(4-methoxybenzyl)acrylamide (**8**)


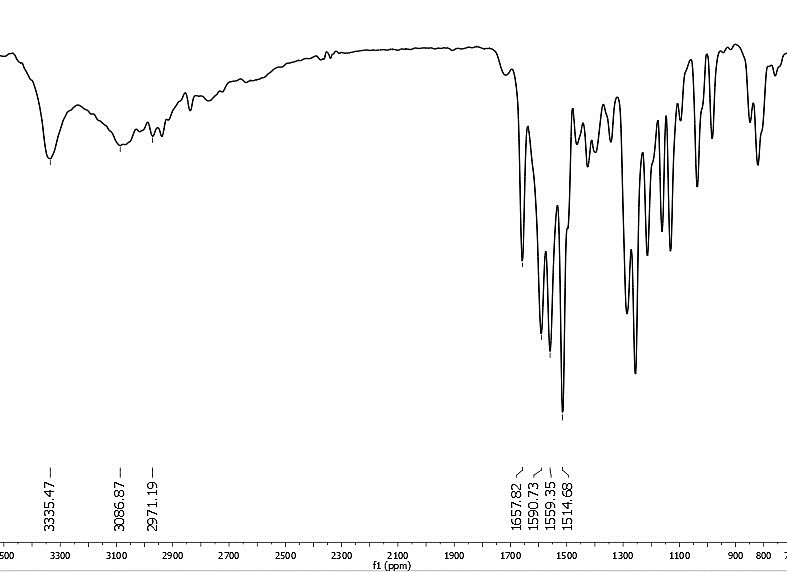


**Figure S24**. IR ʋmax (KBr, cm^-1^) spectrum of (*E*)-N-(4-chlorobenzyl)-3-(3-hydroxy-4-methoxyphenyl)acrylamide (**9**)

**
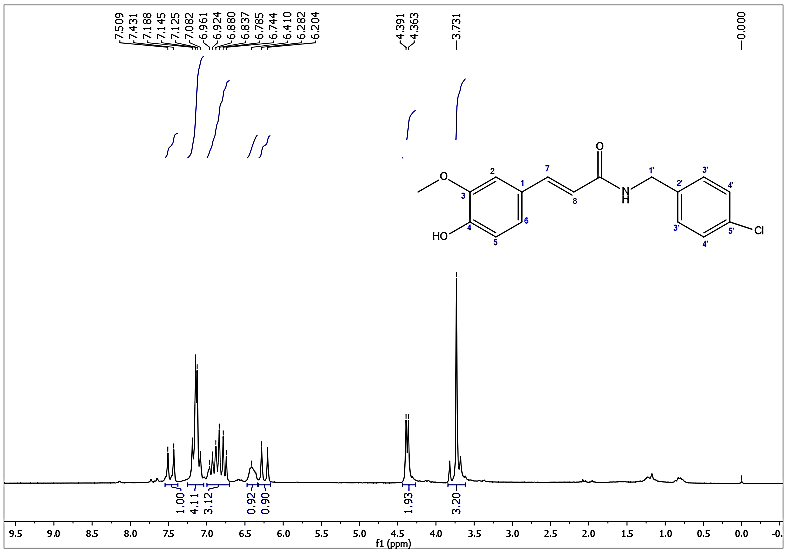
**

**Figure S25**. ^1^H NMR (CdCl_3_, 200 MHz) spectrum of (E)-N-(4-chlorobenzyl)-3-(3-hydroxy-4-methoxyphenyl)acrylamide (**9**)

**
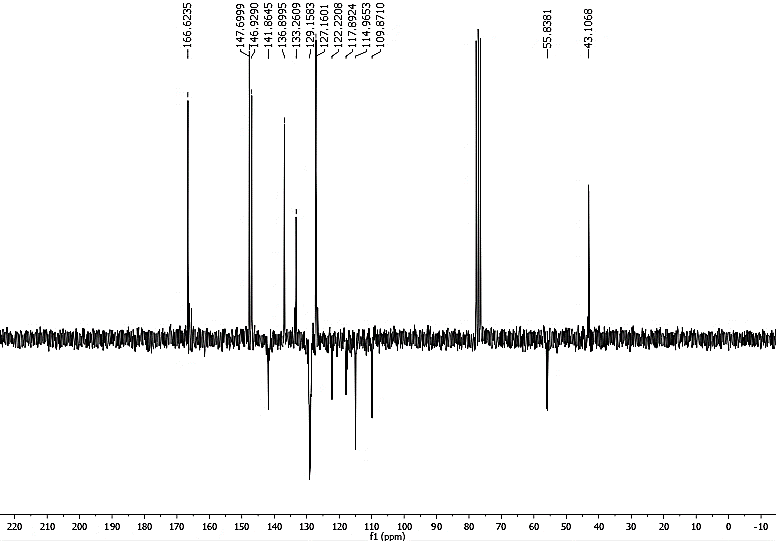
**

**Figure S26**. ^13^C NMR (CdCl_3_, 50 MHz) spectrum of ((*E*)-N-(4-chlorobenzyl)-3-(3-hydroxy-4-methoxyphenyl)acrylamide (**9**)


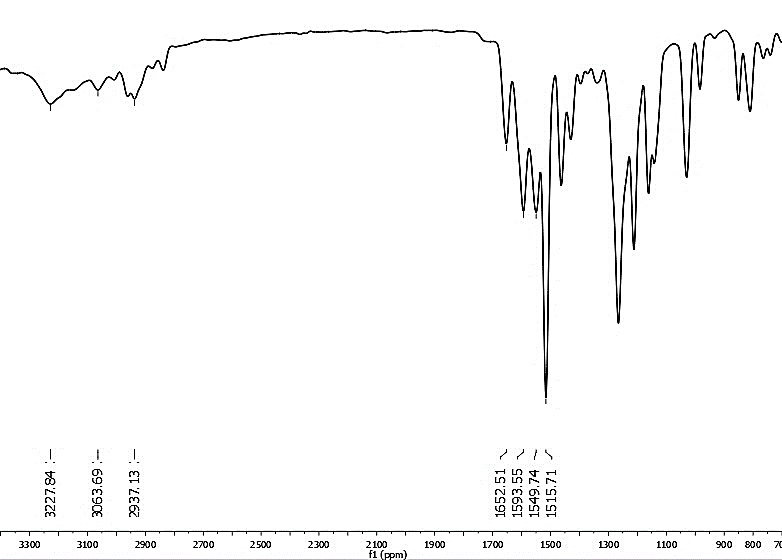


**Figure S27.** IR ʋmax (KBr, cm^-1^) spectrum of (*E*)-N-(3,4-dimethoxybenzyl)-3-(3-hydroxy-4-methoxyphenyl)acrylamide (**10**)


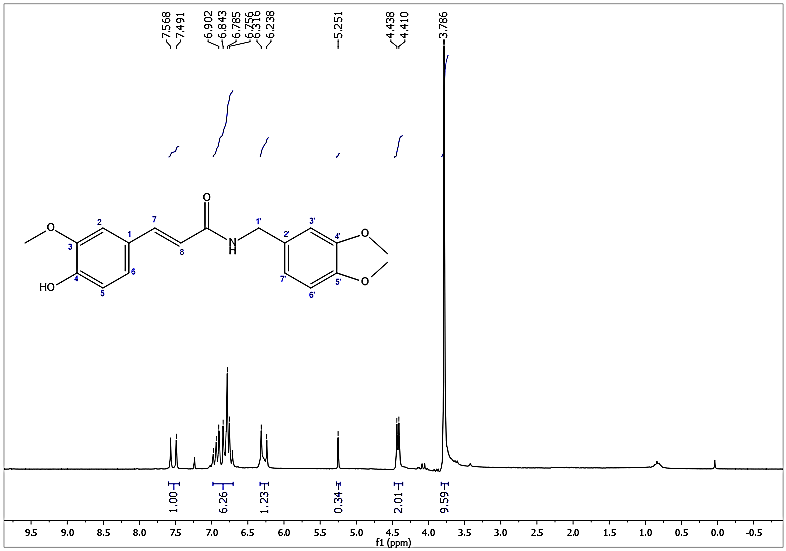


**Figure S28**. ^1^H NMR (CdCl_3_, 200 MHz) spectrum of (E)-N-(3,4-dimethoxybenzyl)-3-(3-hydroxy-4-methoxyphenyl)acrylamide (**10**)


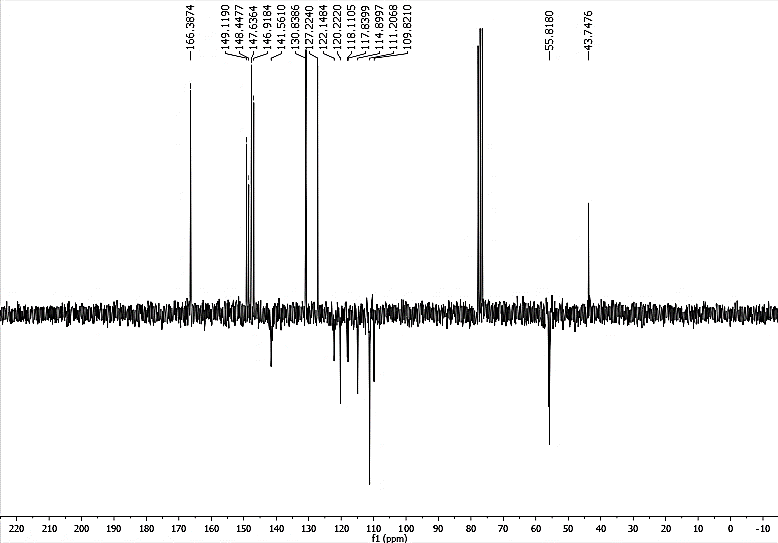


**Figure S29**. ^13^C NMR (CdCl_3_, 50 MHz) spectrum of (E)-N-(3,4-dimethoxybenzyl)-3-(3-hydroxy-4-methoxyphenyl)acrylamide (**10)**

**
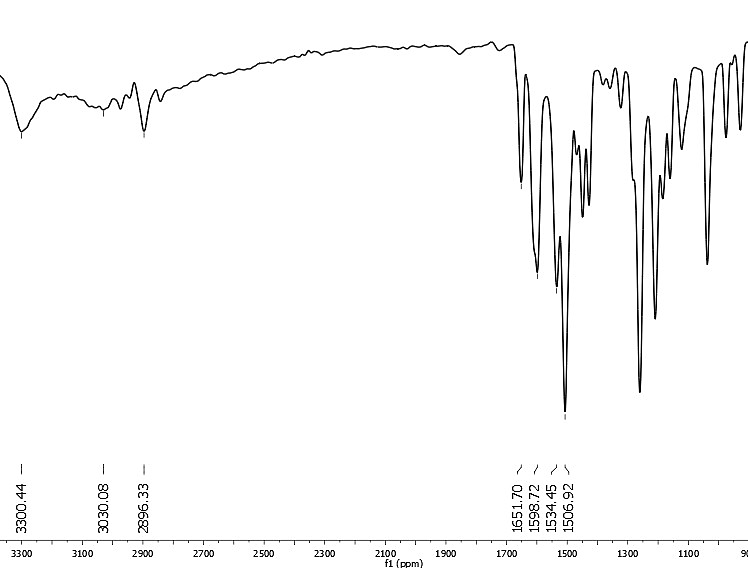
**

**Figure S30.** IR ʋmax (KBr, cm^-1^) spectrum of (*E*)-N-(benzo[d][1,3]dioxol-5-ylmethyl)-3-(3-hydroxy-4-methoxyphenyl) acrylamide (**11**)

**
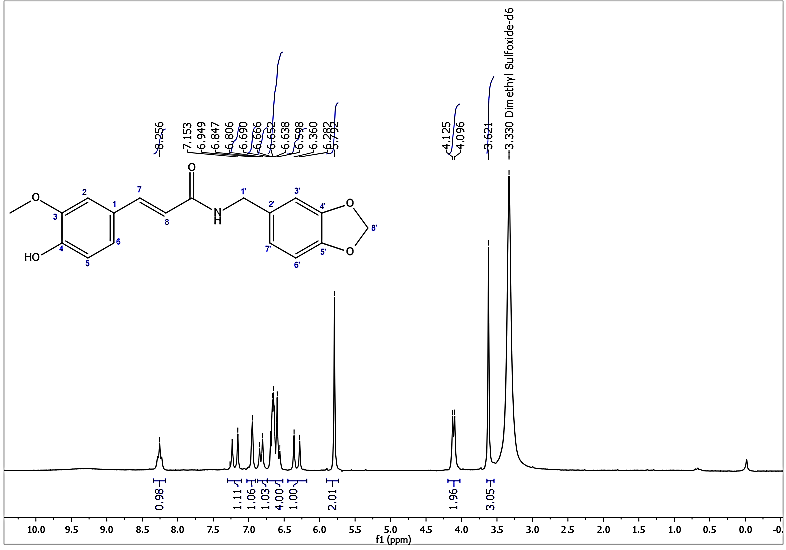
**

**Figure S31**. ^1^H NMR (DMSO-d6, 200 MHz) spectrum of (*E*)-N-(benzo[d][1,3]dioxol-5-ylmethyl)-3-(3-hydroxy-4-methoxyphenyl) acrylamide (**11**)


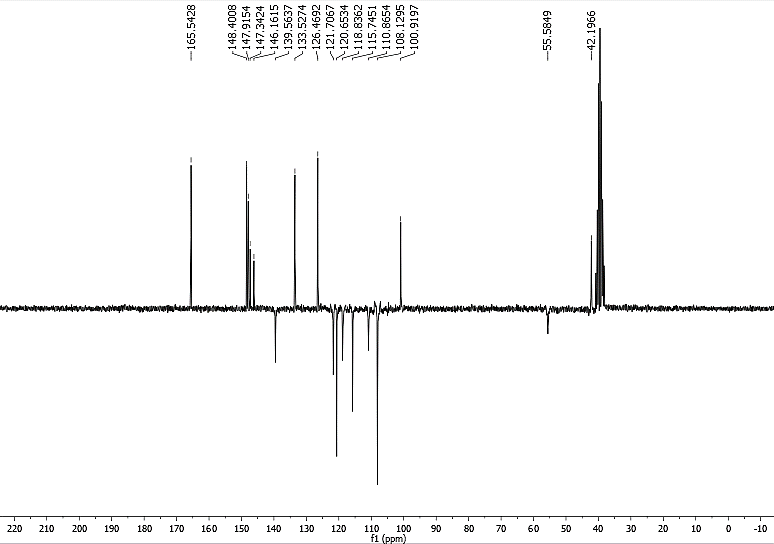


**Figure S32**. ^13^C NMR (DMSO-d6, 50 MHz) spectrum of (*E*)-N-(benzo[d][1,3]dioxol-5-ylmethyl)-3-(3-hydroxy-4-methoxyphenyl) acrylamide (**11**)

1. Molecular modeling

Table S1. Results of docking compounds 10 and 11 to their potential targets.

| Compound | Target | Pose | PLP^(a)^ | Z_PLP^(b)^ | GS^(c)^ | Z_GS^(d)^ | CS^(e)^ | Z_CS^(f)^ | ASP^(g)^ | Z_ASP^(h)^ | Aggregated Z-score |
| --- | --- | --- | --- | --- | --- | --- | --- | --- | --- | --- | --- |
| 10 | KRE6 | 1 | 76.15 | 2.14 | 5.87 | 0.74 | 24.01 | 1.49 | 44.78 | 1.43 | 1.45 |
|  |  | 2 | 70.38 | 1.56 | 8.62 | 0.83 | 19.72 | 0.83 | 47.81 | 1.77 | 1.24 |
|  | XOG1 | 1 | 55.52 | 1.29 | 10.06 | 0.39 | 13.89 | 1.71 | 38.65 | 2.74 | 1.53 |
|  |  | 2 | 56.11 | 1.41 | 15.18 | 0.63 | 11.37 | 0.99 | 34.02 | 1.95 | 1.25 |
|  |  | 3 | 53.75 | 0.95 | 22.2 | 0.97 | 14.16 | 1.78 | 28.92 | 1.09 | 1.2 |
|  | BHSD | 1 | 73.4 | 2.02 | 20.21 | 0.34 | 28.65 | 1.91 | 54.73 | 2.38 | 1.66 |
|  |  | 2 | 71.29 | 1.48 | 36.88 | 0.88 | 25.51 | 0.75 | 50.83 | 1.4 | 1.13 |
|  | CWH41 | 1 | 57.23 | 1.86 | 4.6 | 0.61 | 24.55 | 2.05 | 38.74 | 1.63 | 1.54 |
|  |  | 2 | 54.75 | 1.34 | 6.44 | 0.66 | 20.82 | 1.15 | 37.66 | 1.37 | 1.13 |
|  | SKN2 | 1 | 78.99 | 2.08 | 30.65 | 0.96 | 25.08 | 0.84 | 49.72 | 1.68 | 1.39 |
|  |  | 2 | 75.73 | 1.66 | 27.73 | 0.88 | 24.59 | 0.72 | 44.66 | 0.92 | 1.04 |
|  | BGL22 | 1 | 46.08 | 0.51 | 8.4 | 0.75 | 18.7 | 1.28 | 30.21 | 1.28 | 0.96 |
|  | PHR1 | 1 | 65.57 | 2.24 | 16.01 | -0.01 | 23.22 | 2.1 | 40.92 | 1.89 | 1.55 |
|  |  | 2 | 57.33 | 0.69 | 31.47 | 0.84 | 19.46 | 1.04 | 38.73 | 1.5 | 1.02 |
|  | PHR3 | 1 | 57.21 | 2.49 | -8.43 | -0.21 | 18.41 | 2.08 | 27.83 | 1.39 | 1.44 |
|  |  | 2 | 49.22 | 1.26 | 17.51 | 0.88 | 12.2 | 0.93 | 29.61 | 1.72 | 1.2 |
|  |  | 3 | 49.56 | 1.31 | 26.83 | 1.27 | 12.7 | 1.02 | 24.28 | 0.74 | 1.09 |
|  |  | 4 | 49.78 | 1.34 | 11.71 | 0.64 | 12.76 | 1.03 | 25.8 | 1.02 | 1.01 |
|  | PGA4 | 1 | 59.35 | 2.57 | -4.46 | -0.87 | 17.26 | 2.58 | 31 | 1.25 | 1.38 |
|  |  | 2 | 52.72 | 1.44 | 22.79 | 1.21 | 10.51 | 0.53 | 28.59 | 0.84 | 1.01 |
|  | PGA5 | 1 | 65.48 | 2.29 | 31.49 | 0.88 | 20.7 | 1.92 | 36.38 | 1.42 | 1.63 |
|  | RHO1 | 1 | 73.79 | 1.32 | 41.69 | 1.07 | 28.65 | 1.82 | 31.37 | 1.77 | 1.5 |
|  |  | 2 | 79.85 | 2.28 | 40.89 | 1.02 | 25.82 | 1.16 | 27.4 | 0.6 | 1.26 |
|  | HSP90 | 1 | 67.98 | 2.58 | 24.02 | 1.18 | 27.93 | 2.51 | 28.26 | 1.55 | 1.96 |
|  |  | 2 | 63.29 | 1.75 | 22.11 | 1.06 | 24.03 | 1.48 | 23.86 | 0.33 | 1.15 |
|  | PKC1 | 1 | 68.13 | 2.05 | 3.82 | 0.53 | 24.15 | 1.4 | 29.29 | 0.76 | 1.18 |
|  |  | 2 | 59.26 | 0.56 | 1.46 | 0.42 | 25.97 | 1.99 | 31.28 | 1.32 | 1.07 |
|  | MKC1 | 1 | 69.33 | 1.95 | 26.93 | 1.04 | 25.16 | 1.51 | 33.19 | 2.47 | 1.74 |
|  |  | 2 | 66.85 | 1.36 | 19.23 | 0.73 | 26.03 | 1.98 | 29.72 | 1.32 | 1.35 |
|  | BCK1 | 1 | 60.11 | 1.42 | 24.84 | 0.78 | 22.65 | 1.41 | 26.34 | 2.65 | 1.57 |
|  |  | 2 | 66.64 | 3.53 | -1.89 | -0.62 | 22.44 | 1.3 | 24.82 | 1.87 | 1.52 |
|  | ATC1 | 1 | 53.87 | 1.71 | 12.25 | 0.3 | 18.67 | 1.91 | 37.84 | 1.59 | 1.38 |
|  |  | 2 | 50.75 | 1.31 | -4.94 | 0.26 | 13.13 | 1.25 | 36.12 | 1.41 | 1.06 |
|  | GWT1 | 1 | 79.91 | 3.35 | 26.6 | 0.53 | 22.7 | -0.26 | 38.93 | 1.78 | 1.35 |
|  | MNN1 | 1 | 55.61 | 0.61 | 26.52 | 0.71 | 19.83 | 1.16 | 31.37 | 0.9 | 0.84 |
|  | GSC1 | 1 | 62.94 | 1.22 | 23.42 | 0.23 | 28.7 | 2.79 | 39.7 | 2 | 1.56 |
|  |  | 2 | 67.73 | 2.13 | 18.7 | -0.05 | 26.72 | 2.08 | 38.68 | 1.65 | 1.45 |
| 11 | KRE6 | 1 | 62.99 | 1.19 | 34.72 | 1.24 | 22.3 | 1.72 | 48.95 | 2.26 | 1.6 |
|  |  | 2 | 61.01 | 0.89 | 15.73 | 0.62 | 21.21 | 1.5 | 41.22 | 1.18 | 1.04 |
|  | XOG1 | 1 | 57.23 | 1.81 | 37.54 | 1.26 | 13.03 | 1.15 | 31.6 | 1.11 | 1.33 |
|  |  | 2 | 59.45 | 2.32 | 15.24 | 0.06 | 11.92 | 0.92 | 36.24 | 1.78 | 1.27 |
|  | BHSD | 1 | 78.28 | 2.29 | 35.72 | 0.8 | 30.08 | 2.21 | 60.91 | 2.93 | 2.05 |
|  |  | 2 | 76.01 | 1.84 | 42.68 | 1.45 | 27.24 | 1.31 | 52.63 | 1.2 | 1.45 |
|  |  | 3 | 75.95 | 1.83 | 35.1 | 0.74 | 26.35 | 1.03 | 53 | 1.27 | 1.22 |
|  |  | 4 | 69.18 | 0.49 | 37.06 | 0.92 | 29.21 | 1.93 | 51.99 | 1.06 | 1.1 |
|  | CWH41 | 1 | 60.76 | 1.94 | 17.63 | 0.38 | 17.89 | 0.35 | 35.64 | 0.96 | 0.91 |
|  | SKN2 | 1 | 80.13 | 2.88 | 45.86 | 1.55 | 32.61 | 2.3 | 54.6 | 2.04 | 2.19 |
|  |  | 2 | 73.44 | 1.73 | 32.03 | 0.76 | 22.99 | 0.49 | 56.76 | 2.35 | 1.33 |
|  | BGL22 | 1 | 45.34 | 1.29 | -6.76 | -0.24 | 17.98 | 1.84 | 32.38 | 2.23 | 1.28 |
|  | PHR1 | 1 | 63.75 | 1.65 | 27.76 | 0.46 | 24.32 | 2.43 | 42.4 | 1.8 | 1.58 |
|  |  | 2 | 66.1 | 2 | 32.79 | 0.74 | 19.82 | 1.24 | 37.12 | 0.98 | 1.24 |
|  | PHR3 | 1 | 48.61 | 1.07 | 25.56 | 1.03 | 15.96 | 1.9 | 32.82 | 1.71 | 1.43 |
|  |  | 2 | 53.78 | 1.98 | 1.12 | -0.55 | 15.19 | 1.71 | 31.73 | 1.52 | 1.17 |
|  | PGA4 | 1 | 58.91 | 2.04 | 40.73 | 1.54 | 12.04 | 1.14 | 29.06 | 0.85 | 1.39 |
|  |  | 2 | 48.99 | 0.65 | 19.12 | 0.35 | 14.59 | 1.83 | 31.17 | 1.19 | 1.01 |
|  | PGA5 | 1 | 61.9 | 1.24 | 48.19 | 1.95 | 16.88 | 0.74 | 35.36 | 0.91 | 1.21 |
|  |  | 2 | 62.44 | 1.32 | 29.83 | 0.47 | 20.29 | 1.62 | 38.07 | 1.28 | 1.17 |
|  |  | 3 | 60.94 | 1.11 | 36.21 | 0.99 | 16.26 | 0.58 | 40.27 | 1.57 | 1.06 |
|  | RHO1 | 1 | 76.79 | 1.38 | 56.2 | 1.65 | 30.2 | 2.52 | 32.16 | 1.64 | 1.8 |
|  |  | 2 | 78.46 | 1.62 | 47.66 | 0.94 | 27.54 | 1.86 | 26.74 | 0.18 | 1.15 |
|  |  | 3 | 74.65 | 1.06 | 47.75 | 0.95 | 25.1 | 1.24 | 29.42 | 0.9 | 1.04 |
|  | HSP90 | 1 | 61.14 | 1.67 | 9.44 | -0.43 | 24.03 | 1.55 | 27.17 | 1.14 | 0.98 |
|  | PKC1 | 1 | 63.7 | 1.74 | 18.62 | 1.24 | 20.99 | 0.61 | 31.15 | 1.35 | 1.24 |
|  |  | 2 | 61.34 | 1.22 | 4.88 | 0.3 | 21.84 | 1.13 | 31.62 | 1.51 | 1.04 |
|  | MKC1 | 1 | 66.27 | 1.13 | 43.14 | 2 | 23.97 | 1.47 | 29.66 | 1.22 | 1.45 |
|  | BCK1 | 1 | 59.88 | 2.27 | 22.94 | 0.47 | 21.73 | 1.64 | 25.72 | 2.53 | 1.73 |
|  |  | 2 | 60.08 | 2.34 | 22.88 | 0.47 | 21.63 | 1.58 | 24.34 | 1.84 | 1.56 |
|  | ATC1 | 1 | 55.21 | 1.75 | 12.38 | 0.31 | 10.48 | 0.82 | 38.66 | 1.47 | 1.09 |
|  | GWT1 | 1 | 82.79 | 3.65 | 41.5 | 0.99 | 25.65 | 1.93 | 38.01 | 1.52 | 2.02 |
|  |  | 2 | 70.79 | 1.63 | -3.3 | -0.73 | 24.71 | 1.5 | 38.9 | 1.81 | 1.05 |
|  | MNN1 | 1 | 59.9 | 1.81 | 32.55 | 0.92 | 19.8 | 1.41 | 31.06 | 0.88 | 1.26 |
|  |  | 2 | 58.53 | 1.44 | 21.4 | -0.01 | 20.05 | 1.52 | 32.89 | 1.35 | 1.07 |
|  | GSC1 | 1 | 63.71 | 1.62 | 31.36 | 0.57 | 22.29 | 1.31 | 42.63 | 2.34 | 1.46 |
|  |  | 2 | 65.47 | 2 | 30.06 | 0.44 | 21.72 | 1.03 | 42.37 | 2.27 | 1.43 |

^(a)^ PLP score, ^(b)^ Z-scaled PLP score, ^(c)^ GoldScore score, ^(d)^ Z-scaled GoldScore score, ^(e)^ ChemScore score, ^(f)^ Z-scaled ChemScore score, ^(g)^ ASP score, ^(h)^ Z-scaled ASP score

Table S2. Predicted free energies of binding of compounds 10 and 11 to their potential targets

| Compound | Target | Pose | MM-PBSA Component | | | | | | | ΔG_Total_ |
| --- | --- | --- | --- | --- | --- | --- | --- | --- | --- | --- |
|  |  |  | VD WAALS | EEL | EPB | ENPOLAR | EDISPER | ΔG gas | ΔG solv |  |
| 10 | KRE6 | 1 | -49.16 | -46.41 | 157.26 | -35.54 | 59.56 | -95.56 | 181.28 | 85.71 |
|  |  | 2 | -48.23 | -33.60 | 135.02 | -34.40 | 58.69 | -81.82 | 159.32 | 77.50 |
|  | XOG1 | 1 | -49.26 | -20.38 | 67.12 | -34.53 | 58.59 | -69.64 | 91.17 | 21.54 |
|  |  | 2 | -50.19 | -18.15 | 131.61 | -33.59 | 57.55 | -68.34 | 155.57 | 87.23 |
|  |  | 3 | -42.50 | -3.43 | 105.64 | -31.54 | 53.93 | -45.92 | 128.03 | 82.10 |
|  | BHSD | 1 | -38.23 | 1.66 | 70.39 | -28.38 | 50.70 | -36.57 | 92.71 | 56.14 |
|  |  | 2 | -35.17 | -26.85 | 77.02 | -27.41 | 47.93 | -62.02 | 97.54 | 35.52 |
|  | CWH41 | 1 | -37.18 | -10.29 | 40.60 | -29.18 | 50.35 | -47.46 | 61.77 | 14.30 |
|  |  | 2 | -38.96 | -21.85 | 50.80 | -30.52 | 52.09 | -60.81 | 72.37 | 11.56 |
|  | SKN2 | 1 | -47.89 | -28.65 | 77.27 | -35.17 | 59.05 | -76.54 | 101.14 | 24.60 |
|  |  | 2 | -49.47 | -54.07 | 154.66 | -36.70 | 61.68 | -103.55 | 179.63 | 76.09 |
|  | BGL22 | 1 | -44.76 | -8.97 | 69.57 | -32.10 | 52.19 | -53.73 | 89.66 | 35.94 |
|  | PHR1 | 1 | -34.68 | -7.38 | 94.97 | -24.96 | 45.51 | -42.07 | 115.53 | 73.46 |
|  |  | 2 | -34.79 | 5.07 | 70.70 | -24.89 | 45.56 | -29.72 | 91.36 | 61.65 |
|  | PHR3 | 1 | -38.20 | -23.78 | 45.60 | -29.66 | 50.95 | -61.97 | 66.90 | 4.92 |
|  |  | 2 | -35.44 | -33.67 | 86.93 | -27.87 | 48.13 | -69.10 | 107.19 | 38.08 |
|  |  | 3 | -32.72 | -32.47 | 87.03 | -25.37 | 44.91 | -65.19 | 106.57 | 41.38 |
|  |  | 4 | -34.67 | -10.57 | 92.27 | -26.53 | 46.09 | -45.25 | 111.83 | 66.59 |
|  | PGA4 | 1 | -37.10 | -9.91 | 59.52 | -26.56 | 49.29 | -47.01 | 82.25 | 35.25 |
|  |  | 2 | -32.46 | -35.96 | 60.32 | -26.66 | 47.11 | -68.42 | 80.77 | 12.35 |
|  | PGA5 | 1 | -44.07 | -19.60 | 55.99 | -31.11 | 54.84 | -63.67 | 79.73 | 16.06 |
|  | RHO1 | 1 | -38.91 | -43.74 | 63.81 | -30.57 | 49.18 | -82.65 | 82.43 | -0.22 |
|  |  | 2 | -43.87 | -36.34 | 57.34 | -31.66 | 49.35 | -80.20 | 75.03 | -5.17 |
|  | HSP90 | 1 | -42.33 | -28.66 | 56.58 | -33.41 | 54.13 | -70.99 | 77.31 | 6.32 |
|  |  | 2 | -45.19 | -33.06 | 58.79 | -33.77 | 55.51 | -78.24 | 80.53 | 2.28 |
|  | PKC1 | 1 | -44.53 | -30.66 | 98.09 | -33.38 | 57.26 | -75.19 | 121.97 | 46.78 |
|  |  | 2 | -44.96 | -17.00 | 69.63 | -33.68 | 56.12 | -61.97 | 92.07 | 30.10 |
|  | MKC1 | 1 | -47.11 | -33.98 | 61.07 | -35.25 | 58.99 | -81.09 | 84.81 | 3.72 |
|  |  | 2 | -42.48 | -32.91 | 56.04 | -32.13 | 55.82 | -75.38 | 79.73 | 4.35 |
|  | BCK1 | 1 | -40.94 | -23.59 | 49.86 | -31.93 | 52.61 | -64.54 | 70.54 | 6.01 |
|  |  | 2 | -42.76 | -39.20 | 60.57 | -31.46 | 51.16 | -81.95 | 80.27 | -1.68 |
|  | ATC1 | 1 | -44.70 | -41.78 | 78.02 | -34.96 | 58.11 | -86.48 | 101.16 | 14.68 |
|  |  | 2 | -39.46 | -25.88 | 100.88 | -30.62 | 54.42 | -65.33 | 124.68 | 59.35 |
|  | GWT1 | 1 | -47.36 | 11.01 | 0.00 | -35.16 | 58.33 | -36.35 | 23.17 | -13.18 |
|  | MNN1 | 1 | -37.12 | -25.07 | 54.50 | -28.49 | 50.35 | -62.20 | 76.36 | 14.17 |
|  | GSC1 | 1 | -52.76 | 17.42 | 0.00 | -35.81 | 62.97 | -35.34 | 27.16 | -8.18 |
|  |  | 2 | -46.41 | 12.81 | 0.00 | -34.72 | 59.56 | -33.60 | 24.84 | -8.76 |
| 11 | KRE6 | 1 | -36.77 | -40.69 | 141.81 | -28.53 | 51.95 | -77.46 | 165.23 | 87.77 |
|  |  | 2 | -46.07 | -39.05 | 125.64 | -33.17 | 55.43 | -85.12 | 147.90 | 62.78 |
|  | XOG1 | 1 | -43.62 | -6.13 | 99.46 | -30.18 | 52.93 | -49.75 | 122.22 | 72.48 |
|  |  | 2 | -42.06 | -5.62 | 83.63 | -28.86 | 51.74 | -47.69 | 106.51 | 58.82 |
|  | BHSD | 1 | -30.86 | -21.35 | 92.65 | -23.45 | 42.36 | -52.21 | 111.56 | 59.35 |
|  |  | 2 | -39.61 | -21.90 | 84.51 | -28.78 | 49.52 | -61.51 | 105.26 | 43.75 |
|  |  | 3 | -36.78 | 0.26 | 82.36 | -26.93 | 46.55 | -36.52 | 101.98 | 65.46 |
|  |  | 4 | -38.98 | -28.80 | 62.71 | -28.21 | 50.52 | -67.79 | 85.01 | 17.23 |
|  | CWH41 | 1 | -35.05 | -48.79 | 67.78 | -28.84 | 48.80 | -83.84 | 87.74 | 3.90 |
|  | SKN2 | 1 | -47.08 | -24.35 | 81.49 | -32.94 | 57.47 | -71.43 | 106.03 | 34.60 |
|  |  | 2 | -44.26 | -22.82 | 92.15 | -32.06 | 54.95 | -67.08 | 115.04 | 47.96 |
|  | BGL22 | 1 | -38.57 | -42.44 | 100.84 | -29.93 | 48.68 | -81.00 | 119.58 | 38.58 |
|  | PHR1 | 1 | -34.19 | -0.91 | 68.23 | -24.20 | 42.97 | -35.09 | 87.00 | 51.91 |
|  |  | 2 | -45.16 | 5.75 | 63.98 | -30.51 | 51.45 | -39.42 | 84.92 | 45.50 |
|  | PHR3 | 1 | -35.68 | -15.07 | 48.05 | -26.83 | 45.20 | -50.76 | 66.43 | 15.67 |
|  |  | 2 | -34.18 | -15.85 | 41.50 | -25.05 | 45.42 | -50.03 | 61.87 | 11.84 |
|  | PGA4 | 1 | -38.59 | -18.44 | 144.73 | -26.48 | 44.56 | -57.03 | 162.81 | 105.78 |
|  |  | 2 | -34.41 | -21.12 | 57.65 | -25.18 | 45.19 | -55.53 | 77.66 | 22.13 |
|  | PGA5 | 1 | -33.21 | -20.60 | 52.80 | -25.25 | 44.50 | -53.81 | 72.05 | 18.23 |
|  |  | 2 | -31.52 | -20.49 | 47.47 | -23.56 | 42.57 | -52.01 | 66.48 | 14.48 |
|  |  | 3 | -33.75 | -24.85 | 55.99 | -25.76 | 44.79 | -58.60 | 75.02 | 16.43 |
|  | RHO1 | 1 | -39.38 | -22.73 | 45.56 | -29.08 | 45.54 | -62.11 | 62.02 | -0.09 |
|  |  | 2 | -41.58 | -26.73 | 50.73 | -30.89 | 50.39 | -68.31 | 70.23 | 1.92 |
|  |  | 3 | -41.54 | -39.11 | 60.65 | -29.78 | 50.26 | -80.65 | 81.13 | 0.48 |
|  | HSP90 | 1 | -39.28 | -38.73 | 62.92 | -30.77 | 52.48 | -78.01 | 84.63 | 6.62 |
|  | PKC1 | 1 | -36.75 | -9.77 | 54.00 | -28.54 | 49.49 | -46.53 | 74.94 | 28.42 |
|  |  | 2 | -43.85 | -24.18 | 53.77 | -31.44 | 53.30 | -68.03 | 75.63 | 7.60 |
|  | MKC1 | 1 | -43.08 | -41.34 | 56.30 | -32.71 | 52.83 | -84.42 | 76.42 | -8.00 |
|  | BCK1 | 1 | -41.63 | -38.32 | 73.15 | -31.77 | 48.19 | -79.94 | 89.57 | 9.63 |
|  |  | 2 | -38.81 | -29.54 | 55.46 | -29.73 | 48.62 | -68.35 | 74.34 | 5.99 |
|  | ATC1 | 1 | -31.51 | -16.54 | 89.99 | -24.69 | 45.22 | -48.04 | 110.52 | 62.47 |
|  | GWT1 | 1 | -40.72 | 11.59 | 0.00 | -30.11 | 52.40 | -29.13 | 22.29 | -6.84 |
|  |  | 2 | -46.47 | 13.34 | 0.00 | -32.37 | 55.96 | -33.13 | 23.58 | -9.55 |
|  | MNN1 | 1 | -39.22 | -11.79 | 76.46 | -27.05 | 50.93 | -51.01 | 100.34 | 49.33 |
|  |  | 2 | -38.55 | -22.79 | 81.31 | -26.99 | 51.67 | -61.33 | 106.00 | 44.66 |
|  | GSC1 | 1 | -49.07 | 11.49 | 0.00 | -33.73 | 57.93 | -37.58 | 24.20 | -13.38 |
|  |  | 2 | -45.45 | 12.59 | 0.00 | -33.01 | 57.06 | -32.86 | 24.04 | -8.81 |

**Table S3.** Definitions of the ligand binding pockets

| Target | Reference PDB or Residues list |
| --- | --- |
| KRE6 | 4bpz |
| XOG1 | 4m82 |
| BHSD | 3n9k |
| CWH41 | 5mhf |
| SKN2 | 4bpz |
| BGL22 | 4iih |
| PHR1 | 5oa2 |
| PHR3 | 5fih |
| PGA4 | 5oa2 |
| PGA5 | 5oa6 |
| RHO1 | 6sge |
| HSP90 | 6cjj |
| PKC1 | 4oth |
| MKC1 | 4iz5 |
| BCK1 | 2xik |
| ATC1 | 4ktp |
| GWT1 | L206,G240,L242,F303,L239,T238,F237,R213,W233,F236,N234,F235,G299,L431,S302,V166,Y306,F167,L309,V396,Y392,S164,G163,A388,F165V162,G161,R385,L160,R386,D159,T131,M158,K149,F434,Y229,E228,L135,N438 |
| MNN1 | 6fsn |
| GSC1 | P849,Q1089,K1085,V1214,S1215,L1103,I1104,A1106,D1105,Q1108,N1107,P1200,D1201,H1199,F1154,A1229,D1226,E1225,Y1228,L1223,N1224,I1227,H1196,G1198,G1115,A1157,G1251,K1250,E1159,R1252,I1161,L1195,Y1197,K1265,I1266,F1262,G1181,E1177,F1180 |


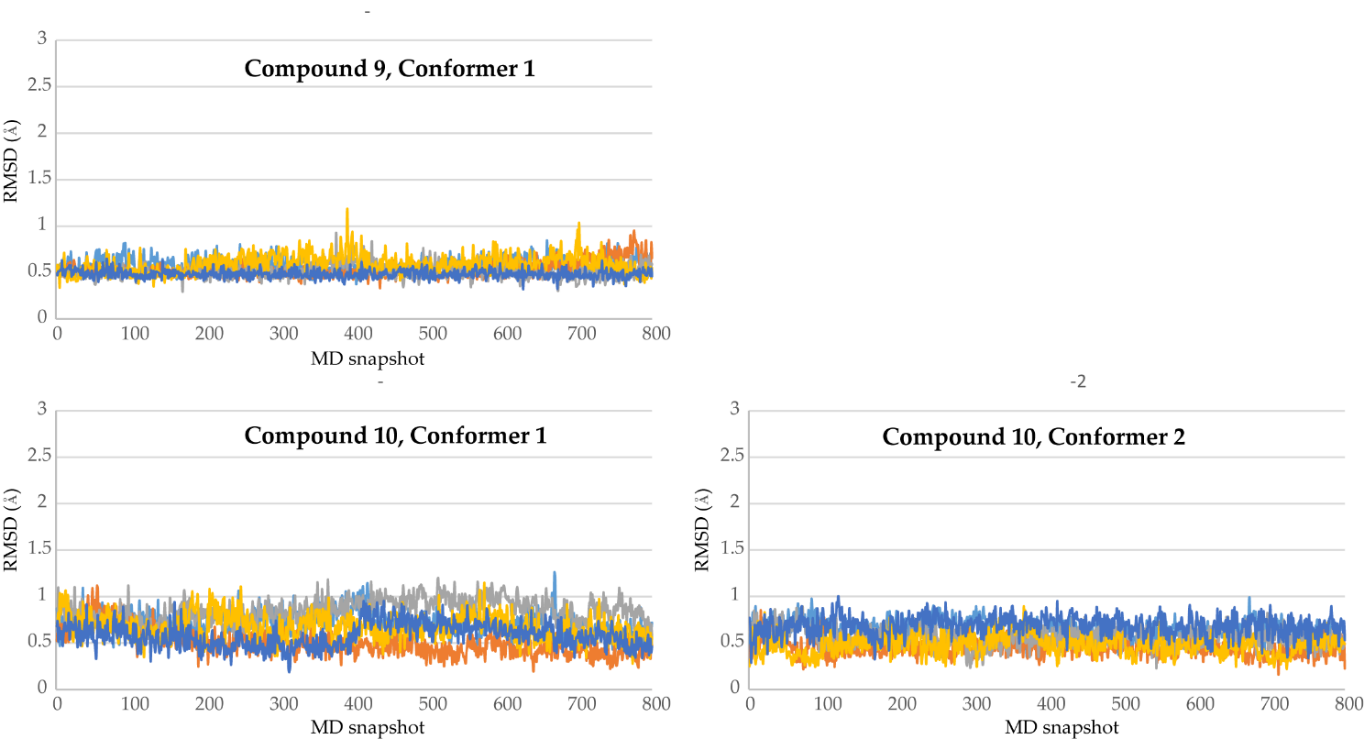


**Figure S33.** RMSD (in Å) of the studied complexes of compounds **10** and **11** with GWT1 along the five 4ns length MD simulations. RMSD is measured for the ligands relative to the docking predicted pose.


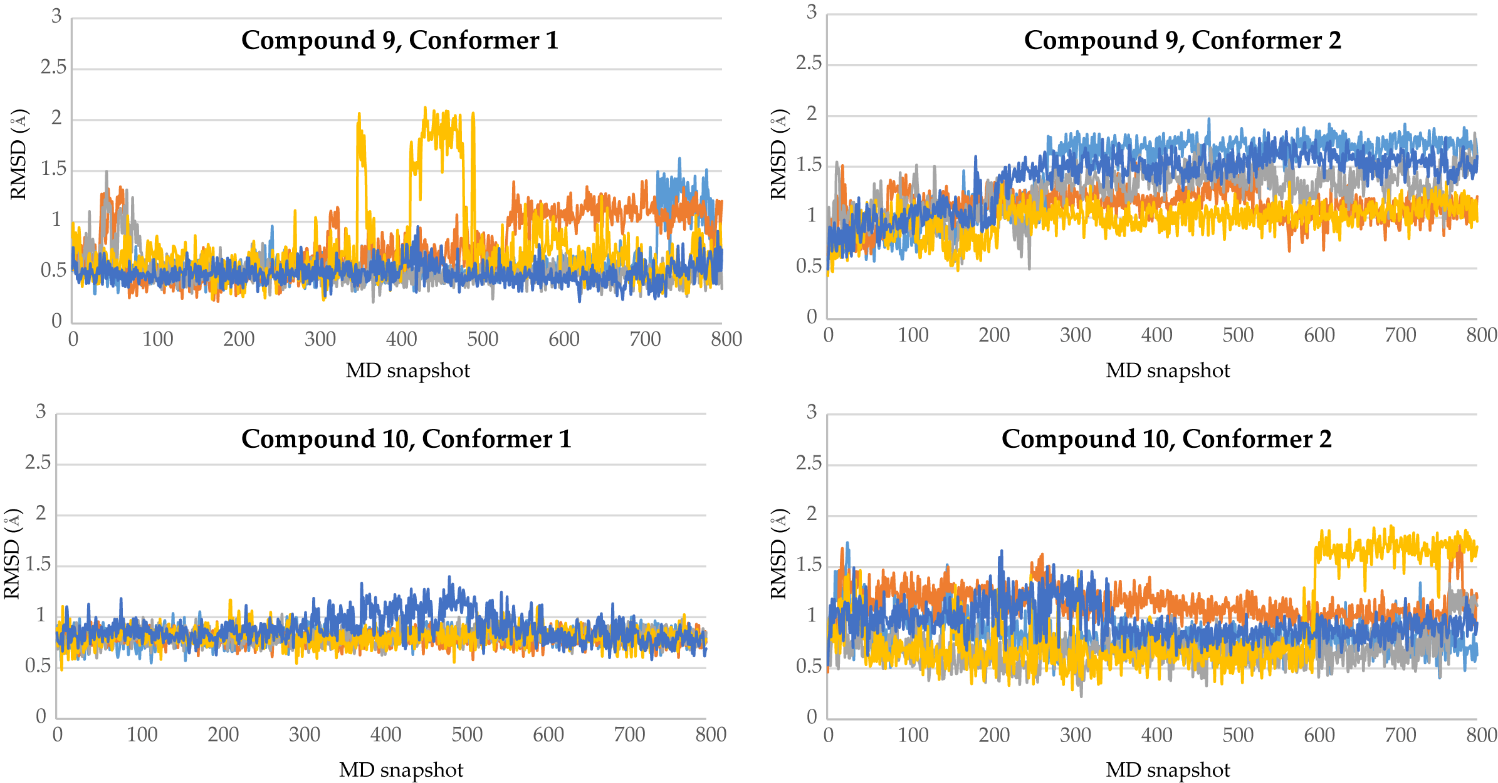


**Figure S34.** RMSD (in Å) of the studied complexes of compounds **10** and **11** with GSC1 along the five 4ns MD simulations performed. RMSD is measured for the ligands relative to the docking predicted pose.

**References**

(1) da Nóbrega, F.; Ozdemir, O.; Nascimento Sousa, S.; Barboza, J.; Turkez, H.; de Sousa, D. Piplartine Analogues and Cytotoxic Evaluation against Glioblastoma. *Molecules* **2018**, *23* (6), 1382. https://doi.org/10.3390/molecules23061382.

(2) Badavath, V. N.; Baysal, I.; Uçar, G.; Mondal, S. K.; Sinha, B. N.; Jayaprakash, V. Monoamine Oxidase Inhibitory Activity of Ferulic Acid Amides: Curcumin-Based Design and Synthesis. *Arch. Pharm. (Weinheim).* **2016**, *349* (1), 9–19. https://doi.org/10.1002/ardp.201500317.

(3) Santos Oliveira, A. J. D. M.; De Castro, R. D.; Pessôa, H. D. L. F.; Wadood, A.; De Sousa, D. P. Amides Derived from Vanillic Acid: Coupling Reactions, Antimicrobial Evaluation, and Molecular Docking. *Biomed Res. Int.* **2019**, *2019*. https://doi.org/10.1155/2019/9209676.

(4) Cardullo, N.; Pulvirenti, L.; Spatafora, C.; Musso, N.; Barresi, V.; Condorelli, D. F.; Tringali, C. Dihydrobenzofuran Neolignanamides: Laccase-Mediated Biomimetic Synthesis and Antiproliferative Activity. *J. Nat. Prod.* **2016**, *79* (8), 2122–2134. https://doi.org/10.1021/acs.jnatprod.6b00577.

(5) Montes, R. C.; Perez, A. L. A. L.; Medeiros, C. I. S.; De Araújo, M. O.; Lima, E. D. O.; Scotti, M. T.; De Sousa, D. P. Synthesis, Antifungal Evaluation and in Silico Study of N-(4-Halobenzyl)Amides. *Molecules* **2016**, *21* (12). https://doi.org/10.3390/molecules21121716.

(6) Chang, Y. H.; Ho, T. Y.; Wu, C. H.; Chen, C. Y.; Huang, H. J.; Tsai, F. J.; Tsai, C. H.; Chen, C. Y. C. Study of AMP-Activated Protein Kinase Agonists by Structure-Based Drug Designing. In *Advanced Materials Research*; Trans Tech Publications Ltd, 2009; Vol. 79–82, pp 2187–2190. https://doi.org/10.4028/www.scientific.net/AMR.79-82.2187.

(7) Jones, E. C. S.; Pyman, F. L. CCCLV. - The Relation between Chemical Constitution and Pungency in Acid Amides. *J. Chem. Soc. Trans.* **1925**, *127* (0), 2588–2598. https://doi.org/10.1039/CT9252702588.
